# Advancing DNA Steganography with Incorporation of Randomness

**DOI:** 10.1002/cbic.202000149

**Published:** 2020-05-28

**Authors:** Meiying Cui, Yixin Zhang

**Affiliations:** ^1^ B CUBE Center for Molecular Bioengineering Technische Universität Dresden Tatzberg 41 01307 Dresden Germany

**Keywords:** DNA steganography, DNA data storage, quantum key distribution-like function, self-destruction

## Abstract

DNA has become a promising candidate as a future data storage medium; this makes DNA steganography indispensable in DNA data security. PCR primers are conventional secret keys in DNA steganography. Brute force testing of different primers will be extremely time consuming, and practically unaffordable when high‐throughput sequencing is used. However, the encrypted information can be sequenced and read once the primers are intercepted. A new steganography approach is needed to make the DNA‐encoded information safer, if not unhackable. Mixing information‐carrying DNA with a partially degenerated DNA library containing single or multiple restriction sites, we have built an additional protective layer that can be removed by desired restriction enzymes as secondary secret keys. As PCR is inevitable for reading DNA‐encrypted information, heating will cause reshuffling and generate endonuclease‐resistant mismatched duplexes, especially for DNA with high sequence diversity. Consequently, with the incorporation of randomness, DNA steganography possesses both quantum key distribution (QKD)‐like function for detecting PCR by an interceptor and a self‐destructive property. It is noteworthy that the background noise generated through the protective layer is independent from any sequencing technology including Sanger and high‐throughput sequencing. With a DNA ink incorporating the steganography, we have shown that the authenticity of a piece of writing can be confirmed only by authorized persons with knowledge of all embedded keys.

## Graphical abstract

**Cryptic clues**: We show DNA steganography using basic molecular biology techniques with a restriction enzyme as a second secret key: Alice encrypts a message and sends it to Bob. Bob needs a combination of keys 1 and 2 to sequence the DNA and retrieve the information; he can also use qPCR to detect any interception by Eve.

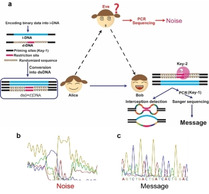

## Introduction

As a novel data‐storage medium, DNA possesses high capacity and longevity, and can be amplified and operated biochemically. The remarkable technological improvement in de novo DNA synthesis has made the use of synthetic DNA as data storage medium feasible.^1^ Recently, encoding digital data into DNA sequences and retrieving the original file without errors have been reported by several research groups.^2^ However, like every other data storage medium, communication with DNA can be intercepted and copied; this has made DNA steganography an important field in DNA data security. DNA steganography provides protective layers to DNA‐encrypted data by mixing dummy DNA sequences with intended information DNA. Clelland et al. have for the first time turned DNA steganography into a reality by hiding information‐carrying DNA in human genomic DNA fragments and spotting them as microdots on filter paper.^3^

Currently, most DNA steganography methods use primer sequences as secret keys that are shared secretly between the sender and recipient to maintain a private information link.^4^ Brute force testing of different primers will be extremely time consuming and practically unaffordable for high‐throughput sequencing. However, the encrypted information can be sequenced and read once the primers are intercepted. The more complex the keys (e. g., more and longer keys), the more secure the encrypted information.^5^ Secret keys can be broken by subjecting the intercepted DNA to intensive analysis. Therefore, in recent years, quantum key distribution (QKD) has been suggested to provide an additional layer of protection for existing encryption algorithms, as its unique feature is the ability of the two communicating users to detect the presence of any third party.^6^ However, currently the rate‐distance limit of QKD hinders large‐scale deployment of QKD networks.^7^

Can we realize QKD‐like function in DNA steganography by using some superior intrinsic properties of DNA over qubits? While QKD is based on the feature that the process of measuring a quantum system disturbs the system, is there a technical analogue of such “measurement” in DNA analysis? Although there are many methods to sequence DNA, for a sample of information‐carrying DNA in small quantity, PCR will be inevitable for the interceptor (“Eve”) to test different keys or key combinations, to amplify the sample for sequencing, as well as to make a copy for the intended recipient (“Bob”). Therefore, if we can make the PCR process generate a disturbance and leave a trace, the communicating users will be able to detect the presence of the third party, analogues to the QKD‐based communication. If the method can be designed based on a physical principle as fundamental as quantum superposition (for QKD), the resulting DNA steganography will be extremely safe, if not unbreakable.

**Scheme 1:**
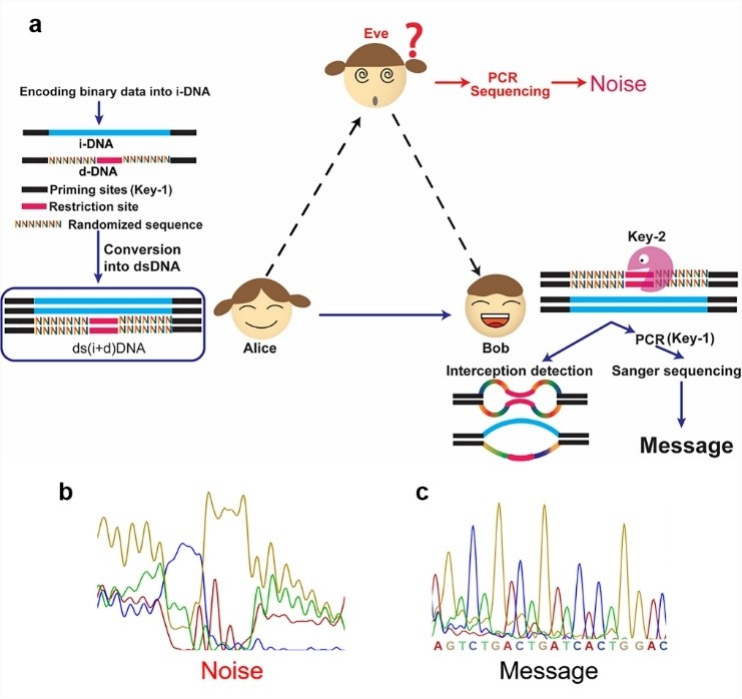
Scheme of the proposed DNA steganography method with incorporated randomness. a) Alice generates an encrypted message and delivers it to Bob. Bob will need to use a combination of key‐1 and key‐2 to be able to sequence the DNA and retrieve the information; he can also use qPCR to detect potential interception (by Eve). Sanger sequencing chromatograms of the message received b) by Eve using the correct primers (key‐1) and c) by Bob using the correct primers (key‐1) after treating the sample with the correct restriction enzyme (key‐2).

Herein, we report the experimental implementation of a DNA steganography method integrating QKD‐like function, a secondary secret key, and a self‐destruct mechanism. Through mixing information‐carrying DNA (i‐DNA) with a partially degenerated DNA library (d‐DNA), the randomness in sequences provides not only an additional mask (e. g., as using genomic DNA) to cover the encrypted information, but also a heat‐induced reshuffling mechanism to generate mismatches during the re‐annealing steps of PCR. It is important to note that the increase in entropy associated with heteroduplex formation through reshuffling is thermodynamically favorable. Thus, without the right combination of restriction enzyme pre‐treatment and primers (Scheme 1), the heating associated with PCR amplification will leave a permanent trace (QKD‐like function) and make the information unreadable, even when the sample is afterwards subjected to the right processing (self‐destruction).

## Results and Discussion

As depicted in Scheme 1, sender “Alice” converts a binary message into a 20‐nt DNA sequence by using a classical substitution cipher.^8^ The conversion algorithm required to translate the binary message into a DNA sequence is not in the scope of this work. The message sequence is flanked by two primer sequences, and the resulting i‐DNA is mixed with a d‐DNA (in large excess). The d‐DNA is of the same length and shares the same primers as i‐DNA, thus can remain as a mask to cover i‐DNA even when the primer information is known to an interceptor (Scheme 1b). The middle 20‐nt region of the d‐DNA contains a 6‐nt restriction site flanked by two 7‐nt randomized sequences, representing a 4^14^ sequence diversity. The single strands are then converted to dsDNA by DNA polymerase. Alice and Bob, but not Eve, share the information regarding primers (key‐1) and restriction sites (key‐2). Receiving the message from Alice, Bob first digests the sample with the desired restriction enzymes (key‐2), then subjects the product to PCR with desired primers (key‐1), and performs sequencing to read the encrypted message (Scheme 1c).

After intercepting the message, Eve cannot read the information without both keys. If she knows the primers (key‐1), the most commonly used secret key, she will amplify the sample by PCR to obtain an adequate amount for sequencing. However, as the information in i‐DNA is masked by d‐DNA, Eve will not be able to distinguish the message from the d‐DNA noise. As shown in Scheme 1b, without the restriction enzyme pre‐treatment (key‐2), the information cannot be read through sequencing.

In addition to key‐1 and key‐2, the steganography also provides a self‐destructive feature and a QKD‐like function. In her attempt to decode the message, Eve is required to amplify the intercepted DNA sample by PCR. However, when amplifying a highly diverse DNA pool, mismatched dsDNA will be generated during the iterated melting and assembling cycles. The mismatches around the restriction sites will affect substrate recognition by DNA endonucleases, diminishing the efficiency of digestion. Therefore, performing PCR prior to enzyme digestion is a self‐destruct process: the mask layer can no longer be removed, as the formation of mismatches cannot be reversed due to the highly diverse d‐DNA in large excess.

**Figure 1:**
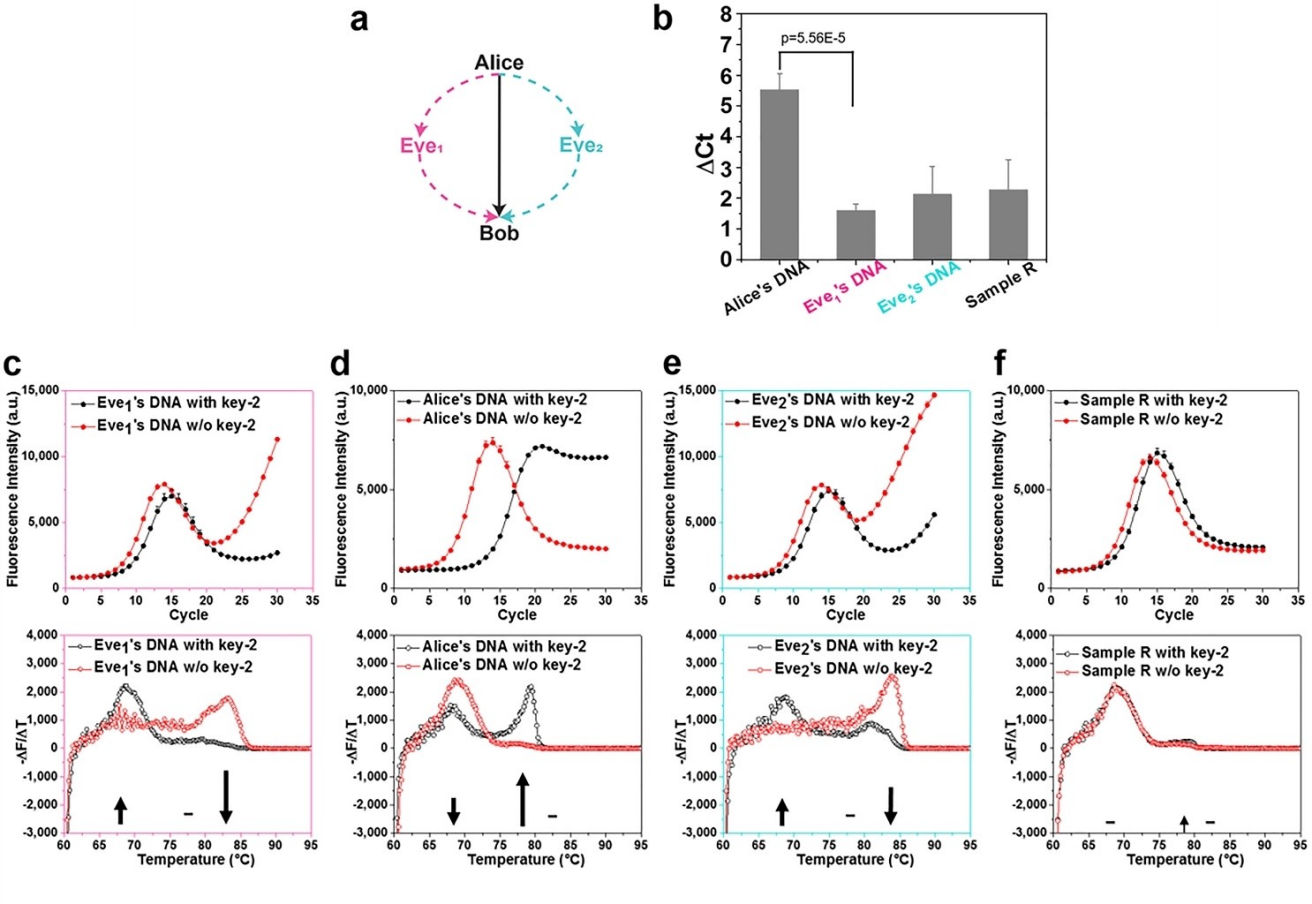
a) Bob can receive three different types of sample: from Eve_1_ (with correct key‐1), the authentic message from Alice, or from Eve_2_ (with a wrong key‐1). b) ΔCt (Ct of treated sample – Ct of untreated sample) of Alice, Eve_1_, and Eve_2_’s DNA, and sample R. All experiments were performed independently five times. Statistical significance was assessed by a paired one‐tail t‐test. t‐value: 15.13, df: 4. c)–f) qPCR amplification curves (top) and melting curves (bottom) for four types of DNA. The size of arrows in melting curves represents the extent of melting peak change in different temperature ranges after key‐2 treatment.

In order to maintain the flow of the communication, after intercepting the message, Eve needs to send the sample to Bob, otherwise Bob will consider it lost and inform Alice. Thus, Bob can use the secret keys not only to decrypt the message, but also to detect whether the message has been “read” before. After performing the restriction enzyme digestion (key‐2), Bob can use quantitative PCR (qPCR) to determine the efficiency of enzyme digestion from the ΔCt value and evaluate the sequence distribution and diversity from the shape of amplification curve and melting curve (Figure 1).^9^ Peaks at a low temperature in the melting curve indicate the presence of less stable heteroduplex; peaks at abnormally high temperature imply higher‐molecular‐weight (MW) structures, which are produced by and accumulated over PCR cycles, especially for templates with high sequence diversity.9a, 10 With the increase in sequence diversity, the qPCR amplification curve will transform gradually from a sigmoid curve to a bell‐shaped curve.^9^ Therefore, by qPCR measurement Bob can judge whether the DNA has been intercepted and subjected to analysis.

By mixing i‐DNA with d‐DNA, the incorporation of randomness aims to give DNA cryptography a second layer of secret key, a self‐destructive feature, and a QKD‐like function. We chose the restriction enzyme SmaI recognition site CCCGGG as the cleavage site in d‐DNA, while i‐DNA and d‐DNA share the same primer sequences (Scheme 1). First, a suitable ratio of i‐DNA and d‐DNA was investigated. i‐DNA was mixed with the d‐DNA at three different ratios (1 : 1, 1 : 10, and 1 : 100). The samples were amplified and subjected to sequencing, simulating the operations that Eve would perform upon intercepting the message and knowing the information of key‐1. As shown in Scheme 1b and Scheme S1 in the Supporting Information, the sequence of i‐DNA can be read when i‐DNA and d‐DNA are mixed at a 1 : 1 ratio, becomes obscure at 1 : 10, and is completely masked at 1 : 100 (Scheme 1b). Therefore, 1 : 100 ratio was used for the following experiments.

Next, we investigated the effect of SmaI treatment on four different types of samples (Figure 1): the 1 : 100 mixtures of i‐DNA and d‐DNA (i+d‐DNA) i) without a PCR pre‐amplification step; with PCR pre‐amplification using ii) correct or iii) wrong primers; and iv) with identical thermocycling in the absence of polymerase and primers (sample R). i+d‐DNA without PCR pre‐amplification represents the authentic message from Alice to Bob; the pre‐amplified sample mimics the message from Eve, (using either correct key‐1 (Eve_1_) or wrong key‐1 (Eve_2_)). The sample R allows us to investigate the effect of heat‐induced reshuffling in the absence of polymerase on restriction enzyme recognition.

Four samples of the same concentration were digested with SmaI and subjected to qPCR with desired primers (key‐1). As shown in Figure 1b, the efficiency of enzyme digestion on R, Eve_1_ and Eve_2_ was remarkably lower than that of Alice's DNA, as evidenced by the ΔCt values. Upon SmaI treatment, i‐DNA, without a cleavable site for the enzyme, became dominant in authentic sample but not in PCR pre‐amplified sample, as shown by the loss of the characteristic bell‐shaped curve (Figure 1d). Moreover, the fluorescence signal started to increase in the later cycles of qPCR in Eve_1_ and Eve_2_. Surprisingly, for Eve_2_, although the template was not amplified due to the use of wrong primers, the presence of polymerase can cause a high end‐point fluorescence signal in the qPCR curve, as DNA sequences with high diversity can intertwine with each other to generate high melting temperature structures (as shown later). In the absence of polymerase, the bell‐shaped curve of sample R wase not affected by the SmaI treatment (Figure 1f).

After enzyme digestion, the melting peak of the authentic sample shifted from 65–70 °C to 75–80 °C, indicative of hetero‐ and homoduplex dsDNA, respectively (Figure 1d). Before enzyme digestion, Eve_1_’s DNA pre‐amplified with correct key‐1 showed a peak at 80–85 °C, indicating high‐MW DNA structures (Figure 1c). Upon enzyme digestion the melting curve redistributed into a remarkable decrease of the main peak at 80–85 °C and a dramatic increase at 65–70 °C. Eve_2_’s DNA also showed a peak at 80–85 °C, which decreased but remained strong after enzyme digestion (Figure 1e). Enzyme digestion caused insignificant increase of the 75–80 °C peak for both Eve_1_ and Eve_2_, as compared to the authentic sample. Therefore, after enzyme digestion, if Bob observes a low ΔCt value, a bell‐shaped qPCR curve, and a melting peak at high temperature (either with or without enzyme digestion), he will be alarmed regarding the interception and possible leakage of key‐1.

After treating the sample with SmaI (key‐2) and PCR amplification (key‐1), Bob will read the message by Sanger sequencing (Scheme 1c). Because enzyme digestion cannot remove the mask of d‐DNA in samples previously subjected to PCR amplification, Bob can only decipher the authentic message from Alice, but not the copy generated by PCR amplification. The interception leaves a trace (QKD‐like function) and destroys the message (self‐destruction). We then investigated the limit of i‐DNA to d‐DNA ratio allowing for enzyme digestion to distinguish i‐DNA from d‐DNA (10 different ratios, Figure S2, S3, and S4). When i‐DNA and d‐DNA are mixed in 1 : 3 ratio, i‐DNA can be clearly read by sequencing without using SmaI, as each base in the degenerated segment at a given position produces only 3/4 of the signal intensity as compared to that from i‐DNA at this position. Therefore, if a digestion product possesses a sequence distribution similar to the 1 : 3 mixture, the samples can be easily read by sequencing. We compared the qPCR curve of 1 : 3 mixture with those from various mixtures after SmaI treatment (Figure S4a, b, and c). At the ratio of 1 : 100 and 1 : 200, but not 1 : 500 and 1 : 1000, the sequence distribution of digested product was close to that of 1 : 3 mixture. Sequencing results also demonstrated that i‐DNA masked in the 1 : 200 sample, but not in the 1 : 500 and 1 : 1000 samples, could be clearly retrieved (Figure S4d, e, and f). At the ratio of 1 : 10, enzymatic digestion can completely remove the d‐DNA cover layer, resulting in qPCR curve shape very close to those of i‐DNA (Figure S2 and S3). This experiment shows the current technical limit for masking i‐DNA with d‐DNA, as further increasing the d‐DNA concentration will make the message unreadable for Bob.

**Figure 2:**
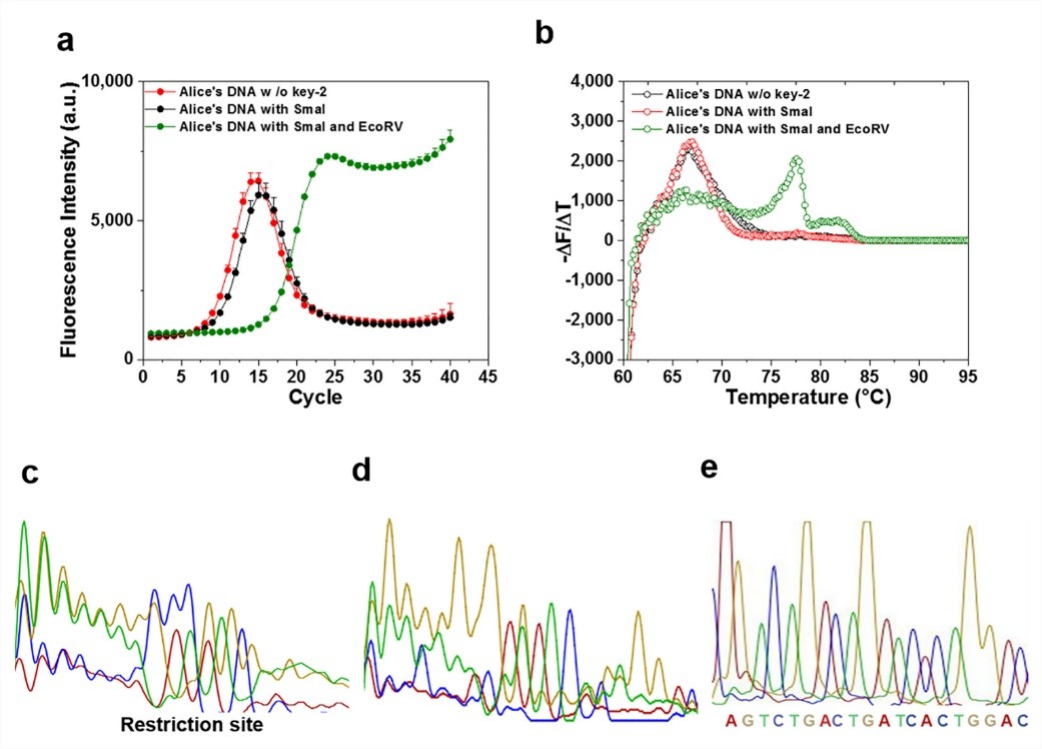
a) qPCR amplification curves and b) melting curves of i‐DNA concealed with two types d‐DNA. Sanger sequencing chromatogram of i‐DNA concealed with two types of d‐DNA c) before key‐2 treatment, d) after SmaI treatment, and e) after SmaI+EcoRV treatment.

Next, we investigated whether other restriction site/enzyme can also be used as key‐2, and whether a combination of two different restriction sites/enzymes can generate a more complex key‐2. We combined two different types of d‐DNA containing SmaI restriction site and EcoRV restriction site and mixed with i‐DNA (50 : 50 : 1) to increase the complexity of the protection layer. The mixture was first treated by SmaI, and followed by EcoRV, then monitored by qPCR and Sanger sequencing (Figure 2). Treatment with SmaI did not change the bell‐shaped amplification curve, while the low ΔCt value of 0.89±0.1 reflected that only half of the mask was removed. Remarkably, an additional digestion step by EcoRV tremendously changed the qPCR curve, indicating a dramatic change in sequence distribution and concentration (ΔCt=3.52±0.17). The message could be fully retrieved by sequencing only after treatments with both enzymes (Figure 2c, e). Only the combination of SmaI and EcoRV can generate a functional key‐2, together with the desired primers (key‐1), for decrypting the concealed information.

In order to prevent Eve from performing brute force testing to discover the key‐2 using a small fraction of intercepted sample, the minimal sample requirement for decoding was investigated. We found that a small amount of DNA as low as 1 fmol can be decoded, as evidenced by the RT‐PCR measurement and Sanger sequencing (Figure S5). However, when we further lowered the amount to 0.1 fmol, endonuclease efficiency was decayed (Figure S5d), as indicated by low ΔCt. Therefore, when the DNA amount is below 1 fmol, it will be challenging for Eve to access i‐DNA with only a small fraction of DNA sample, not mentioning to perform brute force testing by trying different combinations of restriction enzymes.

**Figure 3:**
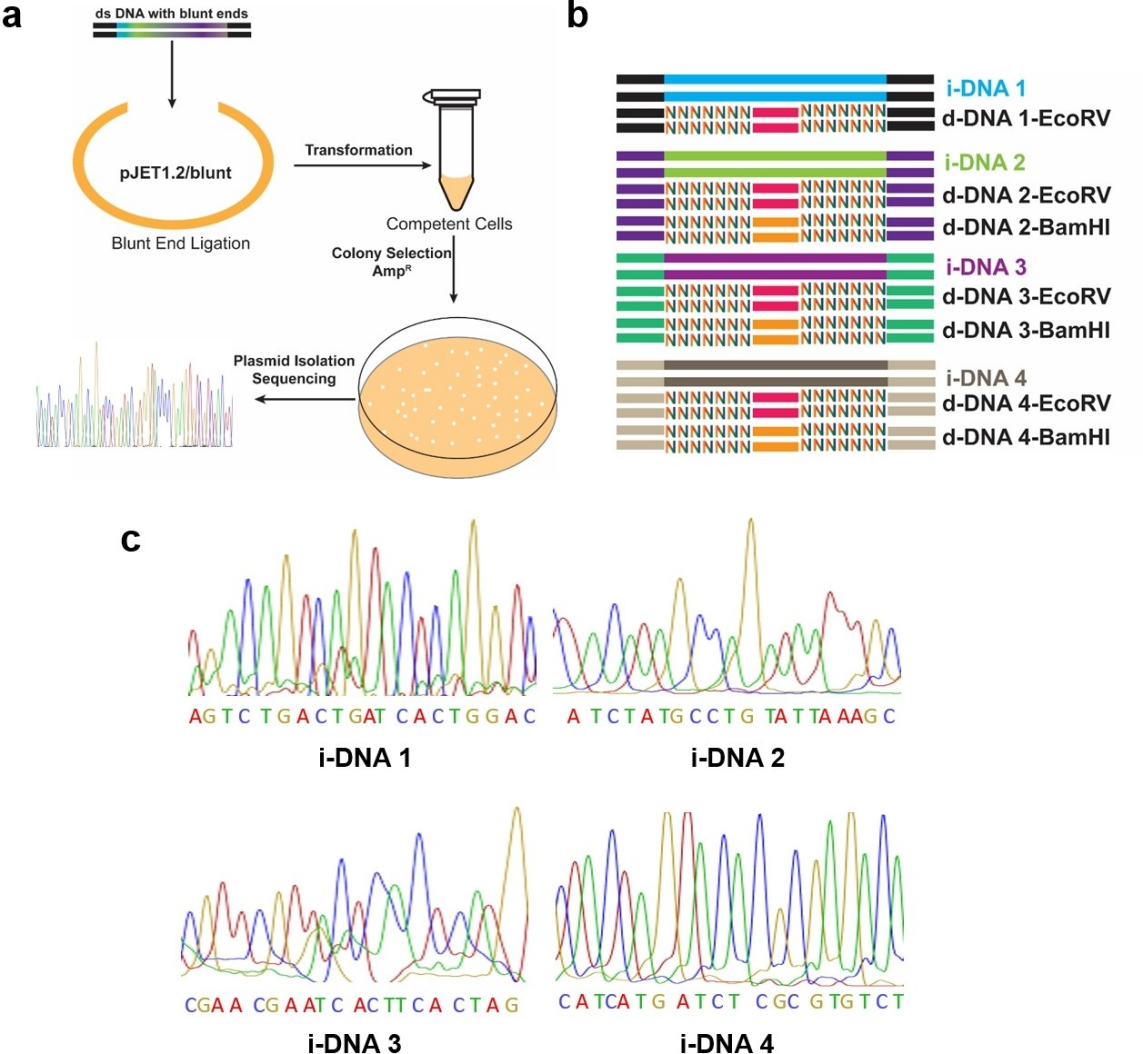
a) Scheme of blunt end ligation‐cloning‐sequencing procedure. b) Four i‐DNAs and corresponding d‐DNAs were mixed to form 0.1 pmol. For each i‐DNA, d‐DNA/i‐DNA=1 : 100. c) Sanger sequencing chromatogram of i‐DNA after decoding (b) with key‐2 and key‐1. The 0.1 pmol DNA mixture was treated with EcoRV and BamHI and subjected to amplification by using individual key‐1 to retrieve respective i‐DNA.

During the course of this study, we received ideas from peer scientists to break the DNA steganography scheme. All steganography methods use PCR primers as keys. However, blunt end ligation and cloning would allow Eve to perform Sanger sequencing without the knowledge about primers (B‐C‐S procedure, Figure 3a). Such potential decryption strategy by Eve has not been investigated in previous DNA steganography studies. Different from simple primer‐based steganography designs, Eve cannot read i‐DNA using the B‐C‐S procedure, as d‐DNA is in large excess. However, the B‐C‐S procedure can still be used to reveal the common primer sequences. We developed a more complex construct as shown in Figure 3b to prevent decryption by using the B‐C‐S procedure.

Four i‐DNAs (i‐DNA 1, i‐DNA 2, i‐DNA 3, and i‐DNA 4) with different primer sequences were mixed with their corresponding d‐DNAs in large excess. The mixed DNA was cloned into the pJET1.2/blunt end cloning vector, transformed to competent DH5α E. Coli, and spread onto LB agar (ampicillin) plate. We have randomly picked 50 colonies, isolated and sequenced the plasmids (Figure S7). Two out of the 50 sequencing chromatograms were either ambiguous or fragmented. The rest 48 sequences could be assigned to d‐DNAs and none of the sequence has appeared more than once. None of the four i‐DNAs was found, as d‐DNAs are in large excess, as compared to i‐DNAs. Only with the specific PCR primers and key‐2, the information encrypted in each i‐DNA can be revealed by Bob (Figures 3c and S6). Decryption by using the B‐C‐S procedure can be further impeded by mixing the sample with excess genomic DNA, which contains no restriction site, or using single stranded i‐DNA to encode the information.

**Figure 4:**
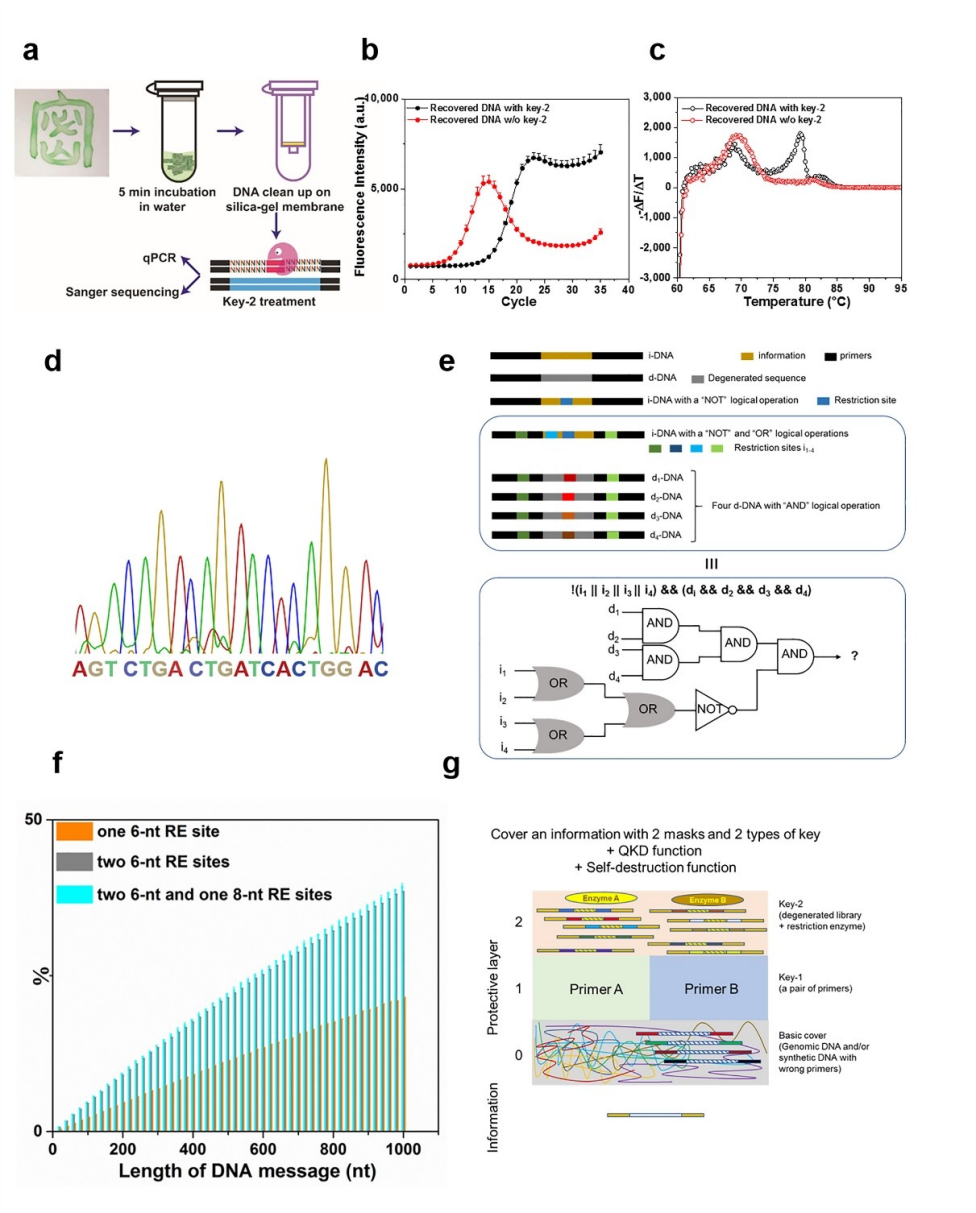
a) Signature of the ancient Chinese character containing DNA steganography and the workflow of i‐DNA recovery. qPCR amplification curves (b) and melting curves (c) of recovered DNA from the signature before and after treatment with key‐2. d) The correct sequence of i‐DNA was revealed by PCR and Sanger sequencing. e) Multiple restriction sites in different d‐DNA sequences can generate “AND” logical operations (e. g., EcoRV and SmaI sites in the d‐DNA); When a restriction site is inserted into any part of i‐DNA, it will create a logical operation of “NOT”. When the interceptor uses the wrong key, the information will be destroyed. Multiple restriction sites in one sequence can generate “OR” logical operations, as any one of the restriction enzymes can cleave the sequence. Therefore, by combining these keys, complex logical operation such as “! (i_1_ || i_2_ || … || i_n_) && (d_1_ && d_2_ && … && d_n_)” can be realized. Where “!”, “||”, “&&” represent NOT, OR, AND logical operations, respectively. i_n_ is a restriction site in i‐DNA, while d_n_ is a restriction site in d‐DNA. f) Probability of DNA sequences (length *N*) that contain the defined restriction site(s), out of 100 000 randomly generated sequences. g) DNA steganography with three layers of masks. Layer 0: dummy DNA as in conventional DNA steganography. Layer 1: correct primers are required to specifically target i‐DNA. Layer 2: degenerated library and corresponding restriction enzyme can protect the message from interceptor with correct primers.

Because of the fast developments in digital and engineering technologies, the traditional function of signature for self‐identification has never been so severely challenged. To demonstrate that the DNA steganography can be used to produce materials for signature, whose authenticity can be confirmed only by people with the knowledge of the secret keys, the mixture of i‐DNA and d‐DNA was added to an ink. As shown in Figure 4a, the Chinese character “secret” (in an ancient seal script) was written on a filter paper with the DNA ink. To analyze the DNA incorporated into the writing, the paper was cut into small pieces and incubated in water. The DNA in solution was then extracted with a DNA purification cartridge, and subjected to the decoding procedure using the two secret keys. As shown in Figure 4b and c, only with the right keys, PCR amplification led to a sample with relatively simple composition, which can be sequenced to reveal the correct information (Figure 4d). In principle, using this methodology, it is possible to further enhance the security of steganography by using multiple secondary keys in order to embed basic logic operators into the DNA‐encrypted message. For example: 1) multiple restriction sites in different d‐DNA sequences can generate “AND” logical operations (e. g., EcoRV and SmaI sites in d‐DNA); 2) inserting restriction site into i‐DNA is equal to a “NOT” operation; 3) multiple restriction sites in one sequence can generate “OR” logical operation. By combining these keys, complex operation can be realized (Figure 4e).

Longer DNA sequence can store more information, as well as to increase the information space exponentially. However, longer sequence will also cause higher probability of a sequence containing certain restriction site(s), such limiting the information space for large data storage. We performed a simulation, to evaluate the probability of a *N*‐nt sequence containing one 6‐bp restriction site, or two 6‐bp restriction sites, or two 6‐bp restriction sites and one 8‐bp restriction site, while *N* is a number between 20 and 1000 (Figure 4f and Supporting Information 2). As expected, increasing *N* or the number of restriction site increases the percentage of randomly generated sequences containing restriction site(s). However, even when *N*=1000 and the number of restriction site is 3, the percentage is <40 %. Although the inclusion of restriction site(s) reduces the information space for *N*‐nt sequence, it does not change the trend of increasing information space when longer DNA is used. For a design with two 6‐bp restriction sites and one 8‐bp restriction site, the growth of information space upon increasing the sequence length outweighs the increase of probability of containing these sites.

## Conclusion

According to quantum mechanics, subatomic particles can simultaneously exist in more than one state, and any attempt to detect the particles’ behavior force the wave function to “collapse” into a defined state, changing the original particles behavior (Heisenberg Principle). The quantum superposition concept, elegantly illustrated by the Schrödinger's cat thought experiment, although sometimes considered as a philosophical paradox, has been successfully used to develop QKD‐based quantum cryptographic communication networks. In essence, if the message is intercepted, the eavesdropper will leave irreversible traces and can be subsequently detected and alert the receivers that a key has been compromised. A solution of DNA molecules does not possess the property of quantum superposition. However, if an attempt of measurement can change its composition and leave a permanent trace, the design of DNA steganography can be considered as a QKD‐like function. When Alice conceals information in a small amount of DNA (e. g., <0.1 pmol), PCR is an inevitable step to prepare the sample for sequencing. The incorporation of restriction sites and randomized domains into the DNA masking sequences will result in interesting properties: the heat‐generated mismatches cannot be recognized by restriction enzymes, and the difference in diversity after enzyme treatment can easily be detected by a number of different methods (e. g., qPCR melting curve, ΔCt, and amplification curve shape). Therefore, without knowing the secondary key (i. e., the nature of the endonuclease) in addition to the correct PCR‐amplification primers (the primary key), the message can neither be analyzed nor be copied by eavesdropper, while the recipient could detect the interception.

As the Chinese saying goes: “While the good climb a foot, the wicked climb ten; it takes constant vigilance to stave off evil.” Throughout human history, encryption and interception/decryption have been two persistent forces fighting against each other (either as “Alice/Bob” vs. “Eve”, or as “the good” vs. “the wicked”), driving inventions and developments in steganography. Therefore, the way to protect information always needs to be discussed in the context of the state‐of‐the‐art technology used to read it. We have shown a DNA steganography utilizing basic molecular biology techniques employing a restriction enzyme as a second secret key. Compared to classical DNA steganography only using primers as key, this approach provides a second layer of mask to cover the information, thus creating massive complexity against brute‐force attack by an eavesdropper. Moreover, this method adds a mechanism to detect interception. Eventually, once the sample is “measured”, it can no longer be processed by enzymes to uncover the information, mimicking a self‐destruct feature. In addition, the information can be further covered by a basic layer (Figure 4g), to prevent simple decryption by using either NGS or the B‐C‐S procedure (Figure 3). For example, the i‐DNAs 2–4 and their corresponding d‐DNAs can be considered either as encrypted information, or as dummy DNAs to cover the information of i‐DNA 1. In the future, when longer DNA constructs are designed for increasing the data storage, nested PCR can be used to improve the detection specificity and sensitivity. CRISPR‐associated Cas proteins can also be implemented to overcome the limitation of available restriction enzymes to further increase the complexity of the steganography.

With the advances in different digital and engineering technologies, it has become increasingly easy to forge a physical object, for example, handwriting or a piece of art, that is indistinguishable from its original. If we incorporate DNA steganography as a signature into materials, it will allow the traditional function of signature to meet the challenges of modern technologies. The self‐destruct function and QKD‐like function can prevent forgers from characterizing and synthesizing the materials, while only the authorized person can confirm the authenticity with the complex keys. In this work, we have demonstrated that a 100‐ to 1000‐fold background noise relative to the encrypted information can be generated through the incorporation of randomness as an additional key, which is independent from any reading technology including both Sanger and high‐throughput sequencing. While increasing the complexity of steganography design can increase both the amount of encrypted information as well as the difficulty to decrypt them (Figure 3 and 4), methods must be constantly evolved in the future in order to meet the challenges of new developments in sequencing and decryption technologies.

## Experimental Section

**Reagents and oligonucleotides**: All oligonucleotides were purchased from IBA life sciences (Göttingen, Germany) at molecular biology grade. Restriction enzymes were purchased from Thermo Fisher Scientific (Waltham, USA). Phusion high‐fidelity DNA polymerase was from New England Biolabs and qPCR master mix was from Quantabio Genomics (Beverly, USA). Agarose gel extraction kit was purchased from Qiagen (Venlo, Netherlands). Sanger sequencing was performed by Eurofins Genomics (Ebensburg, Germany).

**Investigation of suitable ratio between i‐DNA and d‐DNA**: The single‐stranded i‐DNA was mixed with single stranded d‐DNA in 1 : 1, 1 : 10, and 1: 100 ratios. 5 pmol of i+d‐DNA was mixed with excess forward primer and subjected to thermocycling with following protocol. The reaction mixture (50 μL) contained 10x HF buffer, dNTPmix (each 10 nmol), Phusion high‐fidelity polymerase (1 U) and template DNA annealed with forward primer. Thermocycling protocol was 45 s at 98 °C, 5 cycles of 1 min at 55 °C, and 30 s at 72 °C, closing the cycle, final extension for 10 min at 72 °C, and storing at 4 °C. To perform Sanger sequencing, dsDNA product was diluted and subjected to PCR using extended sequencing primers with following protocol: 45 s at 98 °C, then 2 cycles of: 30 s at 98 °C, 1 min at 55 °C, and 30 s at 72 °C, closing the cycle, 18 cycles of: 30 s at 98 °C, 1 min at 65 °C, and 30 s at 72 °C, followed by 10 min at 72 °C, and storing at 4 °C. Then the reaction mixture was loaded on 2 % agarose and 90 V of constant electric field was applied. DNA bands were visualized by a UV transilluminator. The DNA bands of correct size were sliced out and subjected to gel purification using Qiagen gel extraction kit. Purified DNA was mixed with sequencing primer, which annealed to the complementary strand of i‐DNA, and Sanger sequencing was performed.

**Preparation of the authentic message** : 1 : 100 ratio was selected for downstream experiments. Alice sends out 1 pmol of i+d‐DNA.

**PCR pre‐amplification to mimic an eavesdropper's operation**: The 1 : 100 i+d‐DNA pool was amplified using correct primers (Eve_1_) and wrong primers (Eve_2_). The reaction mixture (50 μL) contained 10x HF buffer, dNTPmix (each 10 nmol), Phusion high‐fidelity polymerase (1 U) and template DNA (1 pmol), and 50 pmol of forward and reverse primers. PCR was performed with following protocol: 45 s at 98 °C, 25 cycles of 15 s at 98 °C, 1 min at 55 °C, 30 s at 72 °C, close cycle, final extension for 10 min at 72 °C, then store at 4 °C. Sample R contained neither polymerase nor primers and were incubated in the same way as Eves’ DNA.

**Retrieval of i‐DNA and interception detection by qPCR**: The DNA samples were treated with 10 U of corresponding restriction enzyme in 50 μL reaction volume. Enzyme treatments (key‐2) were performed in thermocycler for 15 min at 37 °C followed by 20 min deactivation at 80 °C. After enzyme treatment, the mixture was diluted 25 times and subjected to qPCR measurement (Thermo Scientific PikoReal Real‐Time PCR System) using following protocol. 10 s at 95 °C, 30 −40 cycles of 15 s at 95 °C, 20 s at 55 °C, 30 s at 72 °C, data acquisition, closing the cycle, 30 s at 72 °C, then starting melting procedure from 60 °C to 90 °C with holding time 1 s, temperature increment after holding 0.2 °C.

**DNA steganography applied as signature**: 1 pmol of Alice's DNA was mixed with water soluble ink. The mixture was then applied on to a cellulose paper (*d*=2 cm) and left to dry. To recover the DNA, the paper was sliced and soaked in 300 μL water and incubated for 5 min. Next, the paper pieces were removed and the solution was purified by a DNA purification based on silica membrane. The resulting DNA was then directly subjected to restriction enzyme digestion and subsequent qPCR analysis as well as Sanger sequencing.

**Blunt end ligation‐cloning‐sequencing (B‐C‐S) procedure** : 0.1 pmol of total DNA mixture containing four types of i‐DNA and seven types of d‐DNA was cloned into 0.05 pmol of pJET1.2/blunt cloning vector (Thermo Scientific, K1231) according to the manufacturer's instruction. The ligation mixture was mixed with 9 fold volume of competent DH5α E.coli cells and incubated on ice for 30 min, then heat shocked at 42 °C for 1 min, then incubated on ice for 5 min. The cells were then allowed to recover in 1 mL liquid LB medium at 37 °C for 1 h. The bacterial solution was pelleted and spread onto LB agar plates containing 100 μg/mL ampicillin and incubated overnight at 37 °C.

On the next day, colonies formed on the plate. 50 colonies were randomly picked from the plate and further cultured individually for 16 h in 5 mL liquid LB‐ampicillin medium at 37 °C. Plasmids were purified (Qiagen, 27104) from 2 mL of bacterial culture and subjected to sequencing with pJET1.2 forward primer.

## Conflict of interest

The authors declare no conflict of interest.

## Supporting information

As a service to our authors and readers, this journal provides supporting information supplied by the authors. Such materials are peer reviewed and may be re‐organized for online delivery, but are not copy‐edited or typeset. Technical support issues arising from supporting information (other than missing files) should be addressed to the authors.

Supplementary
