# Supplementary material for: Advancing DNA Steganography with Incorporation of Randomness
Source: Chembiochem. 2020 May 28;21(17):2503–11. doi: 10.1002/cbic.202000149 (PMC7497043; doi:10.1002/cbic.202000149)
Supplement: Supplementary file 1 — Supplementary [file CBIC-21-2503-s001.pdf]

ChemBioChem

Supporting Information

Advancing DNA Steganography with Incorporation of Randomness

Meiying Cui and Yixin Zhang*© 2020 The Authors. Published by Wiley-VCH Verlag GmbH & Co. KGaA.
This is an open access article under the terms of the Creative Commons Attribution License, which permits use, distribution and reproduction in any medium, provided the original work is properly cited.

Supporting Information

1. Supporting figures

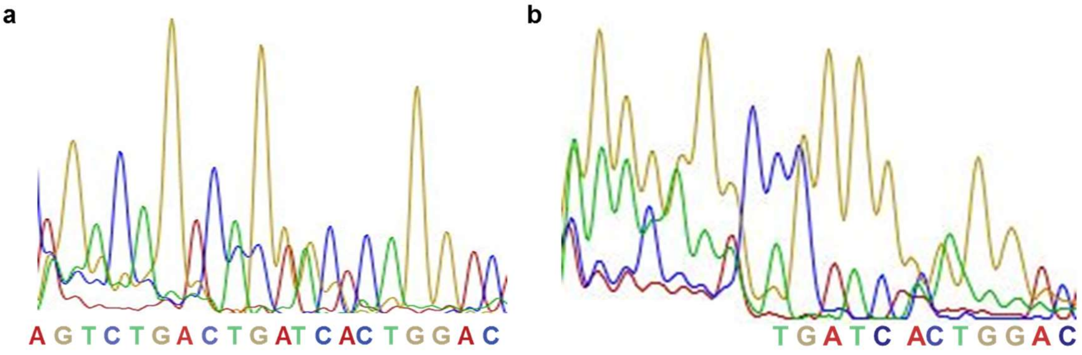

Figure S1. Sanger sequencing chromatogram of i+d-DNA at the ratio of 1:1 (a) and 1:10 (b).

a

Ratio	A	B	C	D	E	F	G	H	I	J	K
i-DNA: d-DNA	Only d-DNA	1:1000	1:500	1:200	1:100	1:10	1:1	2:1	5:1	10:1	Only i-DNA

b

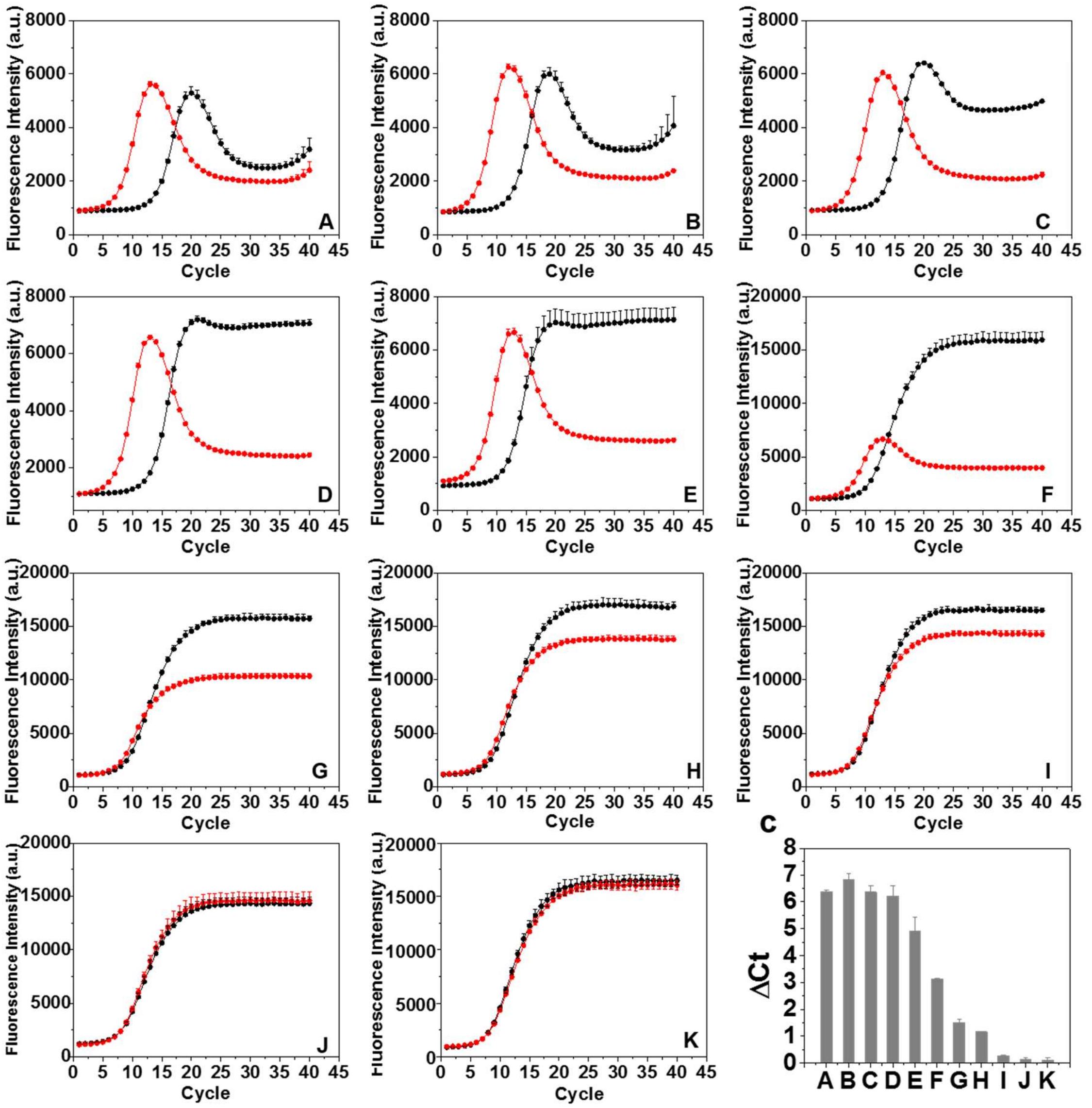

Figure S2. (a) i-DNA and d-DNA was mixed in ten different ratios in the total amount of 1 pmol. (b) qPCR amplification curves of all ratios before (red) and after (key-2) treatment. (c) ΔCt (Ct of sample after key-2 treatment-Ct of sample before Key-2 treatment) values of DNA in ten ratios (A-K).

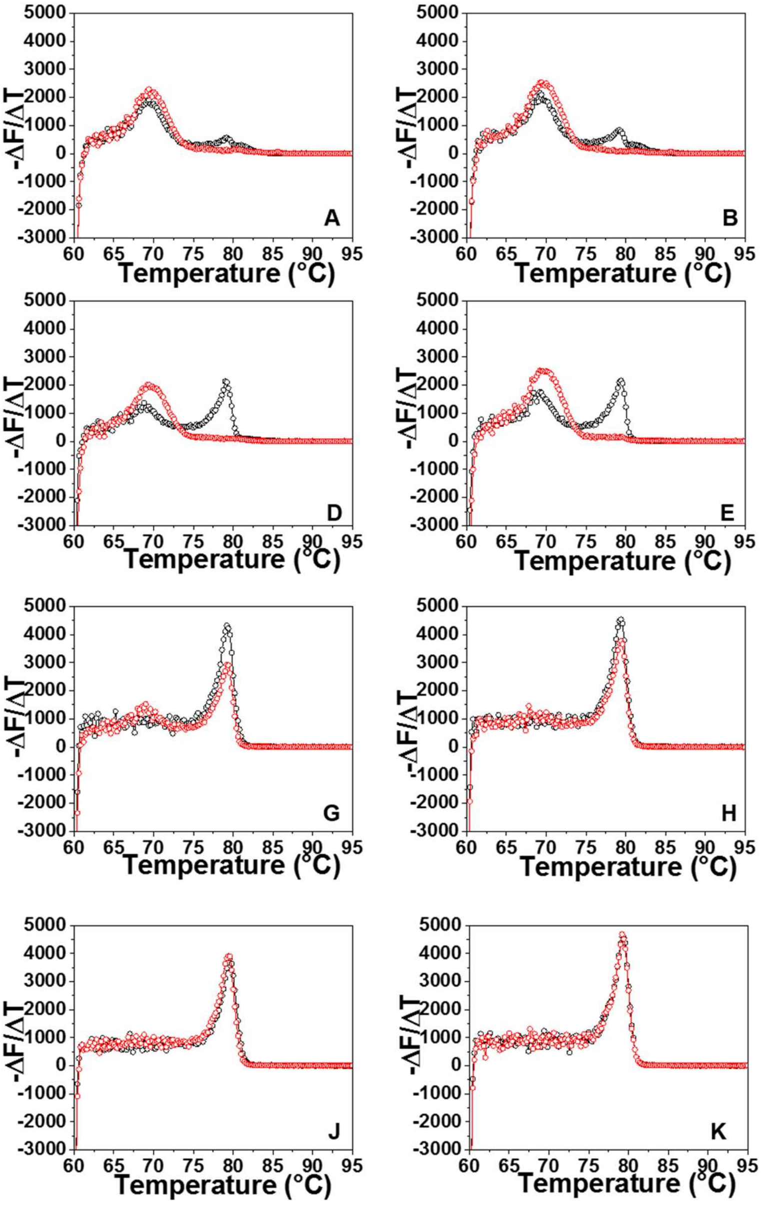

Figure S3. qPCR melting curves of DNA samples (A-K). Red: before key-2 treatment. Blank: after key-2 treatment.

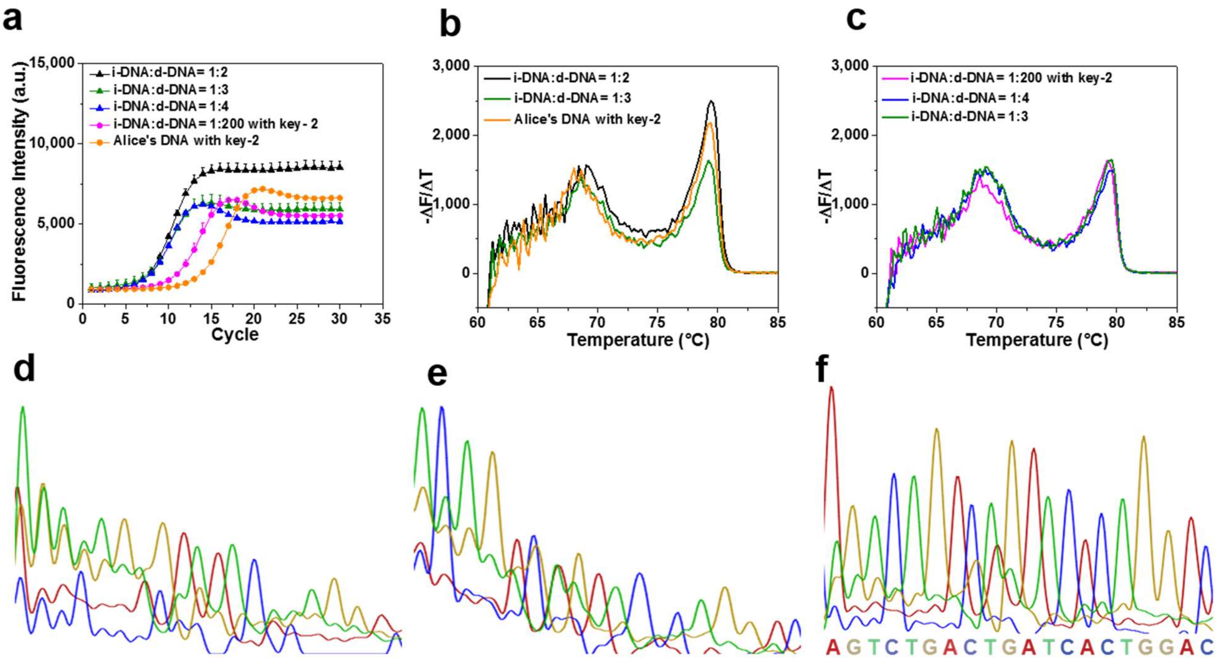

Figure S4. Comparison of key-2 treated i+d-DNA with untreated i+d-DNA at ratio of 1:2, 1:3, and 1:4 via qPCR amplification curve (a) and melting curve (b and c). Sanger sequencing chromatogram of i+d-DNA at the ratio of 1:1000 (d), 1:500 (e), and 1:200 (f) after key-2 treatment.

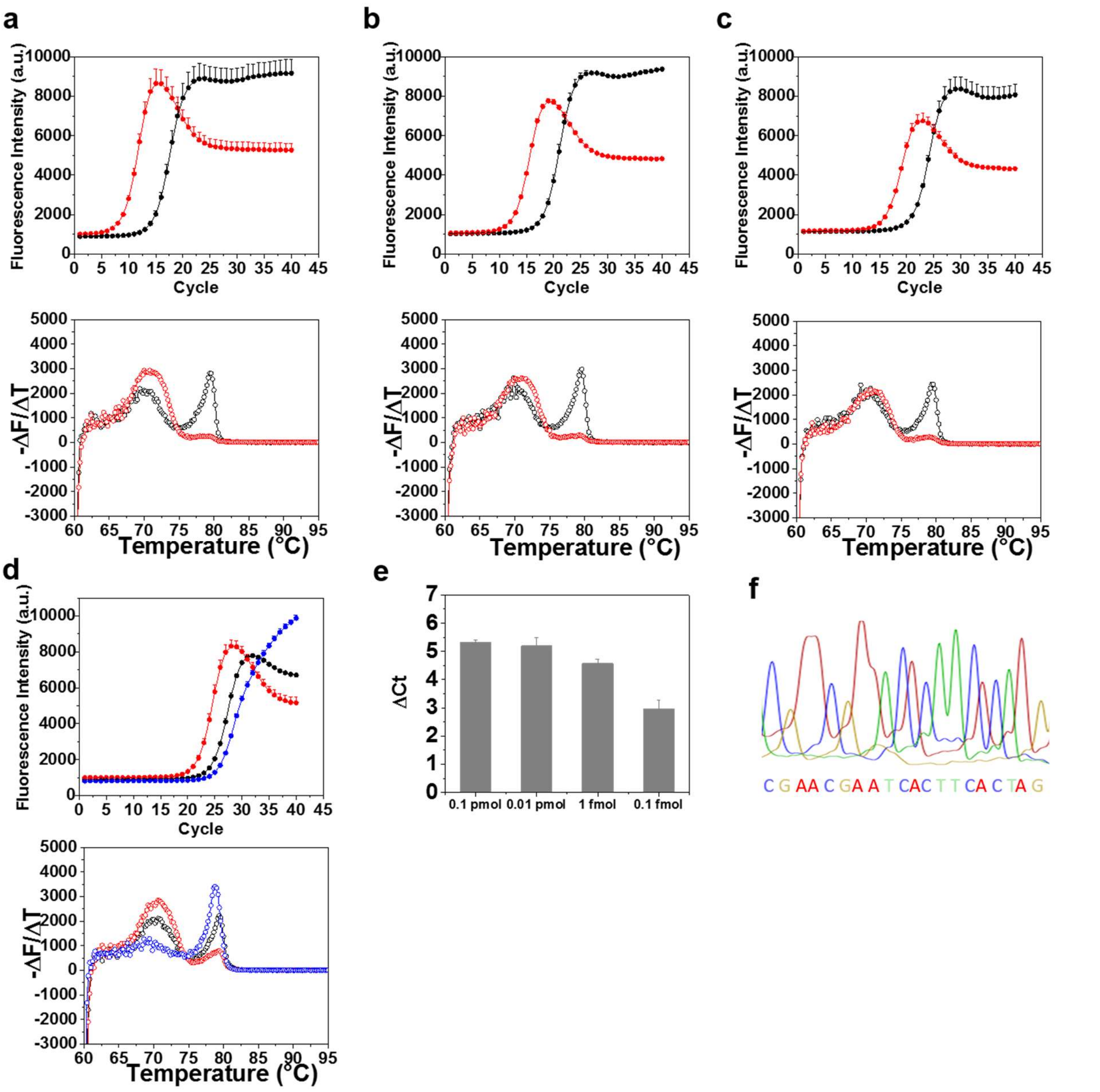

Figure S5. Evaluation of the method on different amount of template DNA. (a) 0.1 pmol, (b) 0.01 pmol, (c) 1 fmol, (d) 0.1 fmol. Red: DNA before key-2 treatment. Black: DNA after key-2 treatment. Blue: Non-templated control with the same primer concentration (blue). (e) ΔCt of DNAs of different amount before and after key-2 treatment. (f) Sanger sequencing chromatogram of i-DNA retrieved from 1 fmol i-DNA: d-DNA (1:100) after key-2 and key-1 treatment.

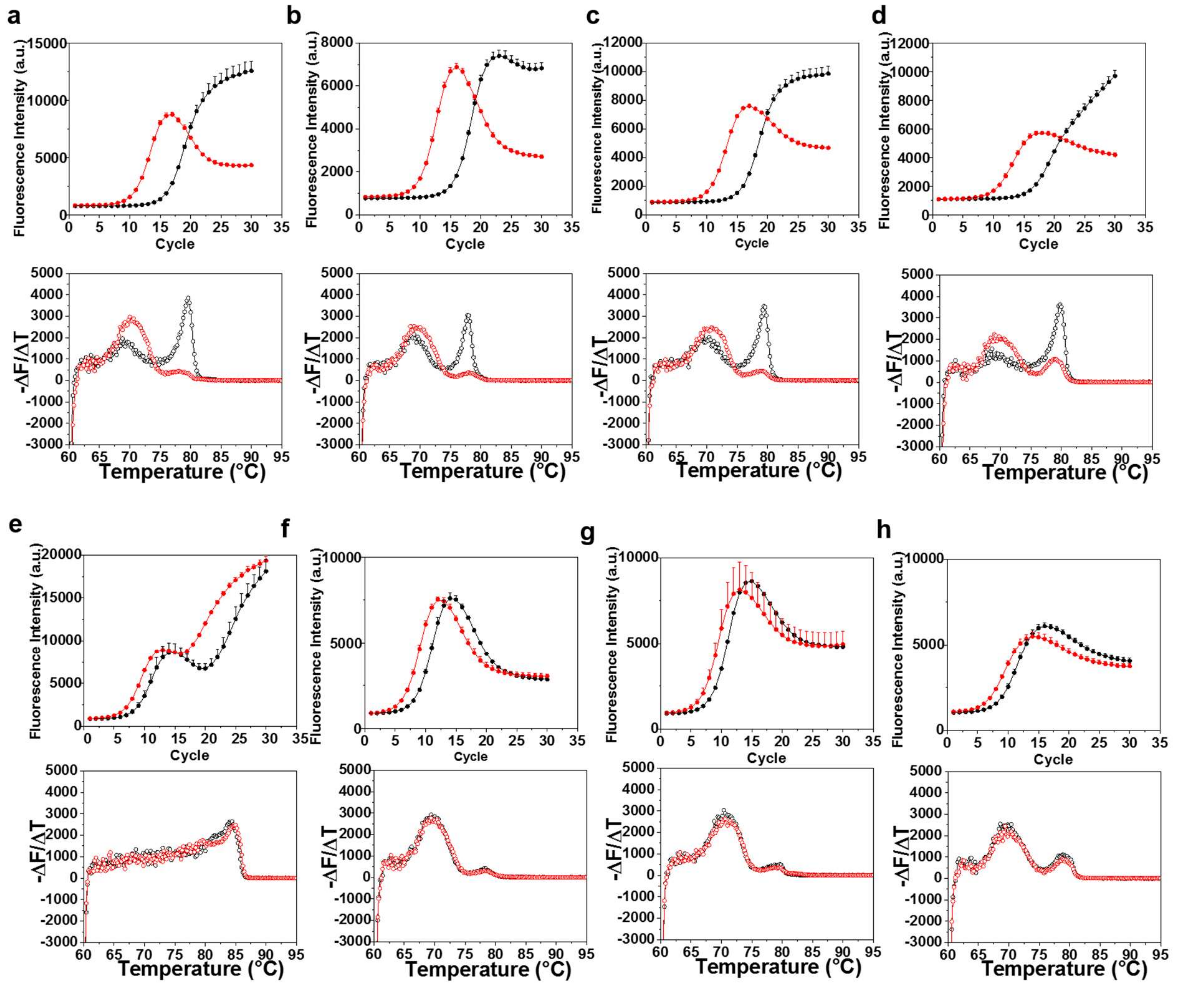

Figure S6. Four i-DNAs and corresponding d-DNAs were mixed to form 0.1 pmol. For each i-DNA, d-DNA: i-DNA=1:100. (a-d) qPCR measurement before (red) and after (black) key-2 treatment (EcoRV and BamHI) using respective key-1 for each i-DNA. (a) i-DNA 1, (b) i-DNA 2, (c) i-DNA 3, and (d) i-DNA 4. (e-h) qPCR measurement of Eve's DNA before (red) and after (black) key-2 treatment using respective key-1 for each i-DNA. (e) i-DNA 1, (f) i-DNA 2, (g) i-DNA 3, and (h) i-DNA 4.

d-DNA 1-EcoRV

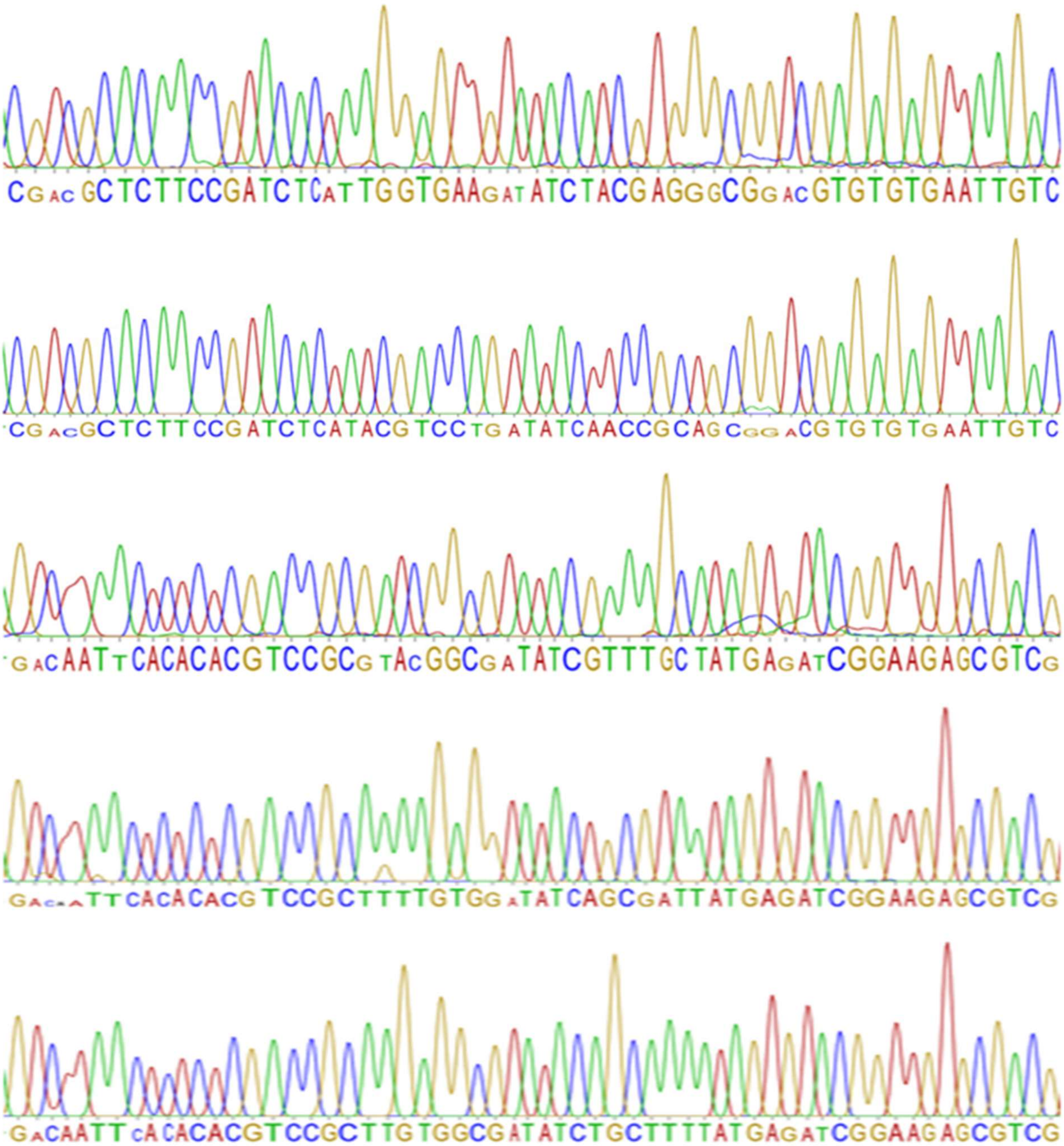
CGACGCTCTTCCGATCTCATTGGTGAAGATATCTACGAGGGCGGACGTGTGTGAATTGTC
CGACGCTCTTCCGATCTCATACGTCCTGATATCAACCGCAGCGGACGTGTGTGAATTGTC
GACAATTCACACACGTCCGCGTACGGCGATATCGTTTGCTATGAGATCGGAAGAGCGTCG
GACAATTCACACACGTCCGCTTTGTGGATATCAGCGATTATGAGATCGGAAGAGCGTCG
GACAATTCACACACGTCCGCTTGTGGCGATATCTGCTTTTATGAGATCGGAAGAGCGTCG

d-DNA 2-BamHI

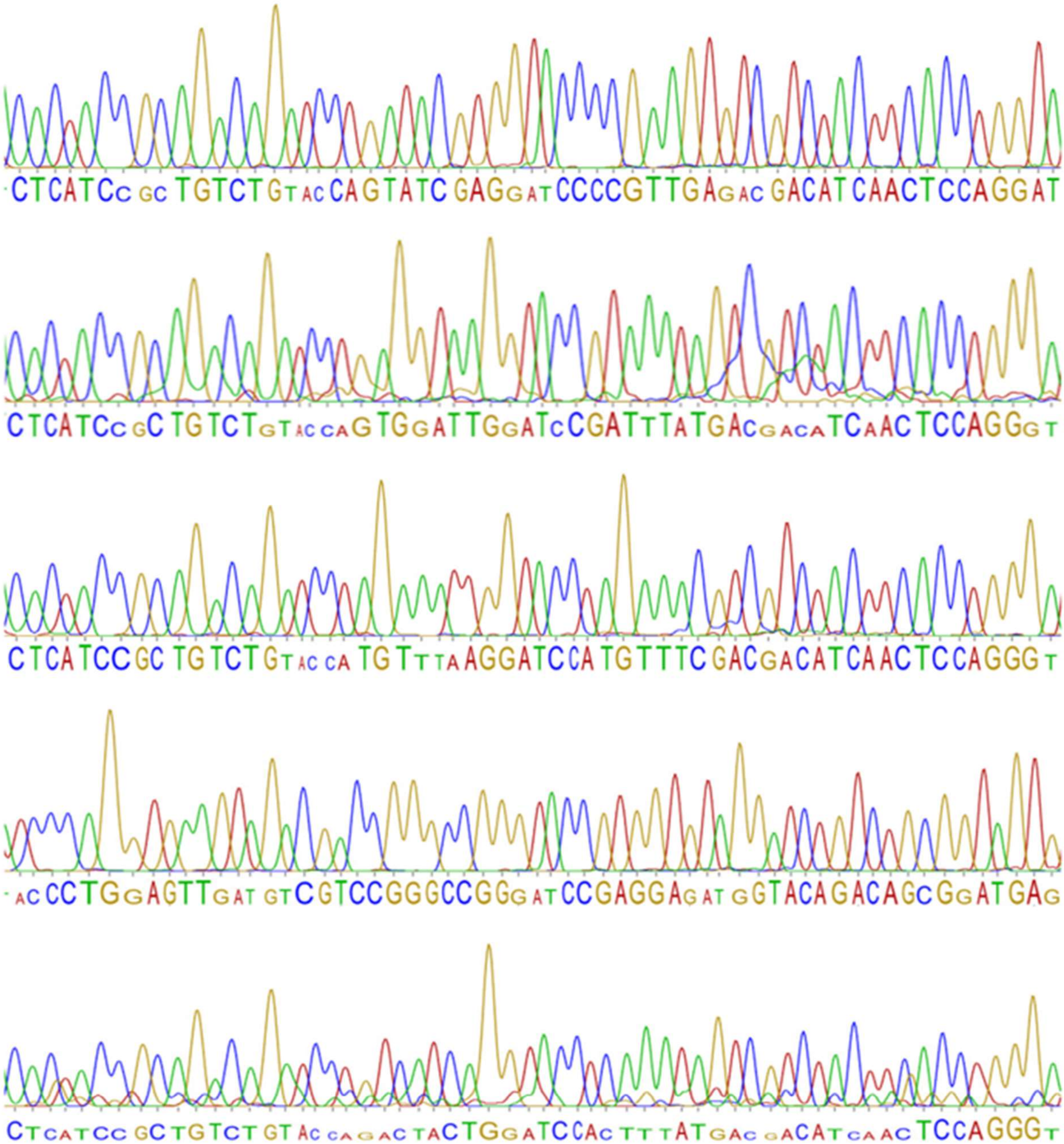
CTCATCCGCTGTCTGTACCAGTATCGAGGATCCCCGTTGAGACGACATCAACTCCAGGAT
CTCATCCGCTGTCTGTACCAGTGGATTGGATCCGATTTATGACGACATCAACTCCAGGGT
CTCATCCGCTGTCTGTACCATGTTTAAGGATCCATGTTTCGACGACATCAACTCCAGGGT
ACCCTGGAGTTGATGTCGTCCGGGCCGGGATCCGAGGAGATGGTACAGACAGCGGATGAG
CTCATCCGCTGTCTGTACCAGACTACTGGATCCACTTTATGACGACATCAACTCCAGGGT
ACCCTGGAGTTGATGTCGTCAGCTTTAGGATCCACGTCACTGGTACAGACAGCGGATGAG

d-DNA 2-EcoRV

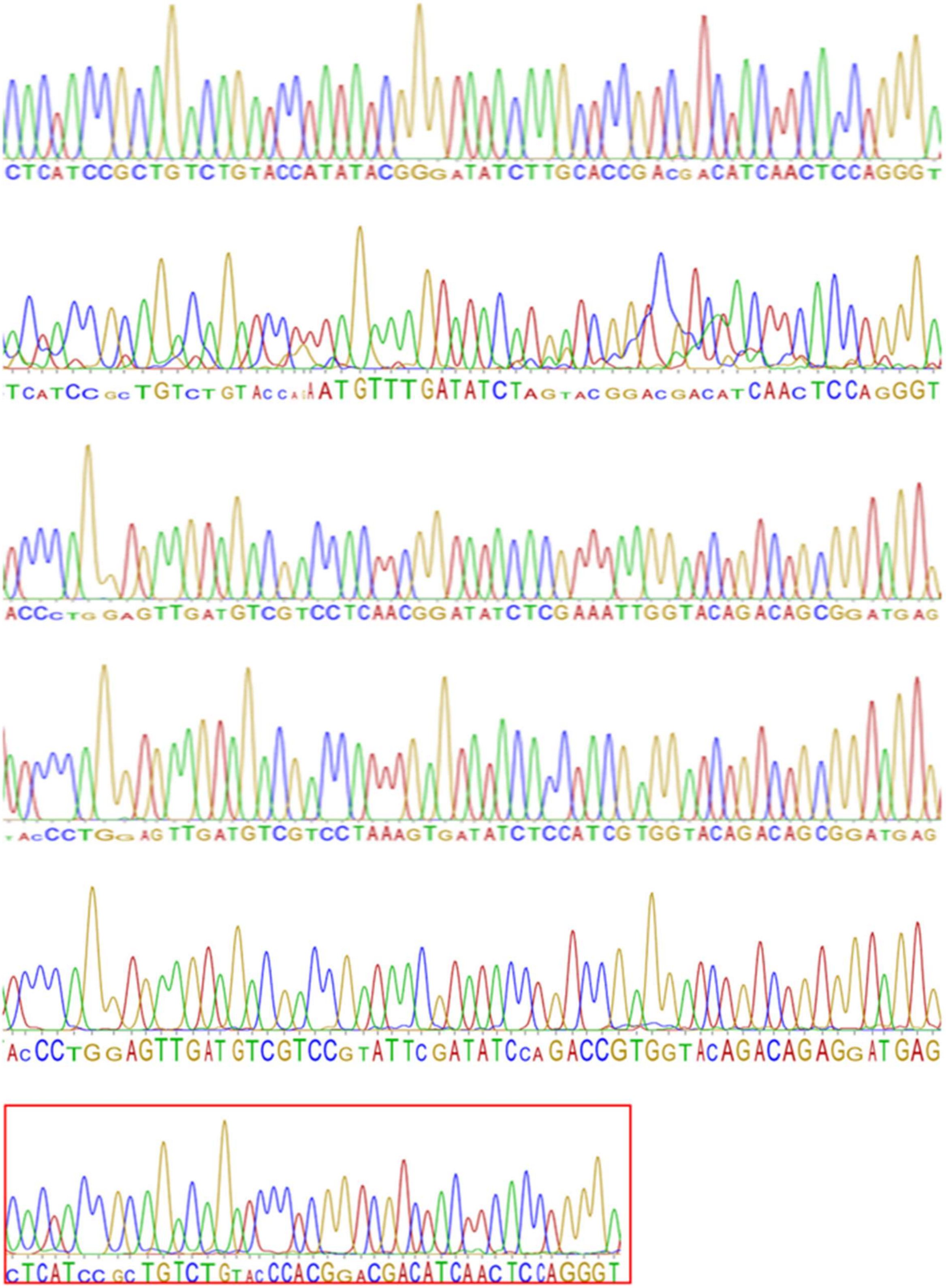
CTCATCCGCTGTCTGTACCATATACGGGATATCTTGCACCGACGACATCAACTCCAGGGT
TCATCCGCTGTCTGTACCAAATGTTTGATATCTAGTACGGACGACATCAACTCCAGGGT
ACCCTGGAGTTGATGTCGTCCTCAACGGATATCTCGAAATTGGTACAGACAGCGGATGAG
ACCCTGGAGTTGATGTCGTCCTAAAGTGATATCTCCATCGTGGTACAGACAGCGGATGAG
ACCCTGGAGTTGATGTCGTCCGTATTCGATATCCAGACCGTGGTACAGACAGAGGATGAG
CTCATCCGCTGTCTGTACCCACGGACGACATCAACTCCAGGGT

d-DNA 3-BamHI

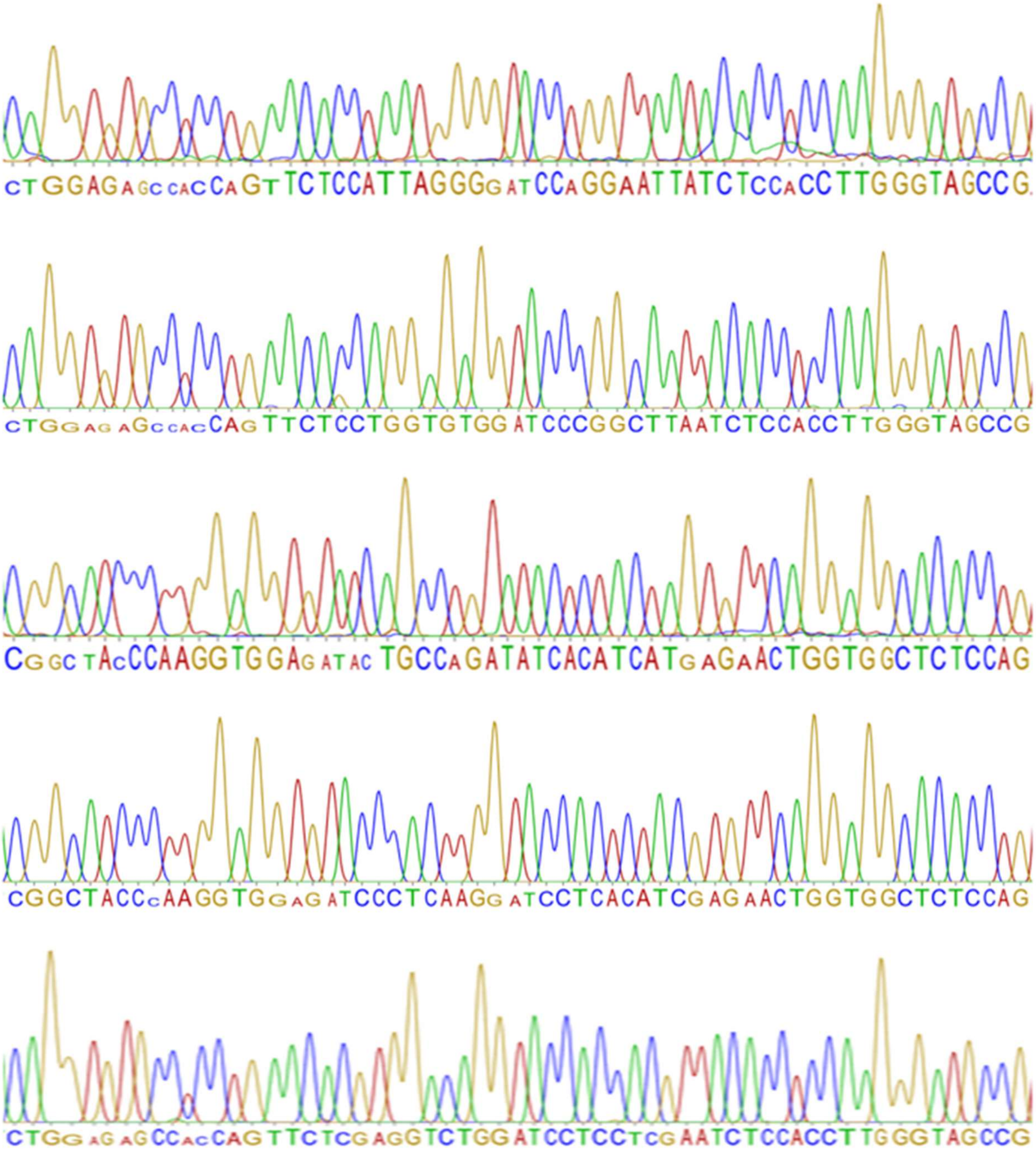
CTGGAGAGCCACCAGTTCTCCATTAGGGGATCCAGGAATTATCTCCACCTTGGGTAGCCG
CTGGAGAGCCACCAGTTCTCCTGGTGTGGATCCCGGCTTAATCTCCACCTTGGGTAGCCG
CGGCTACCAAGGTGGAGATACTGCCAGATATCACATCATGAGAACTGGTGGCTCTCCAG
CGGCTACCCAAGGTGGAGATCCCTCAAGGATCCTCACATCGAGAACTGGTGGCTCTCCAG
CTGGAGAGCCACCAGTTCTCGAGGTCTGGATCCTCCTCGAATCTCCACCTTGGGTAGCCG

d-DNA 3-EcoRV

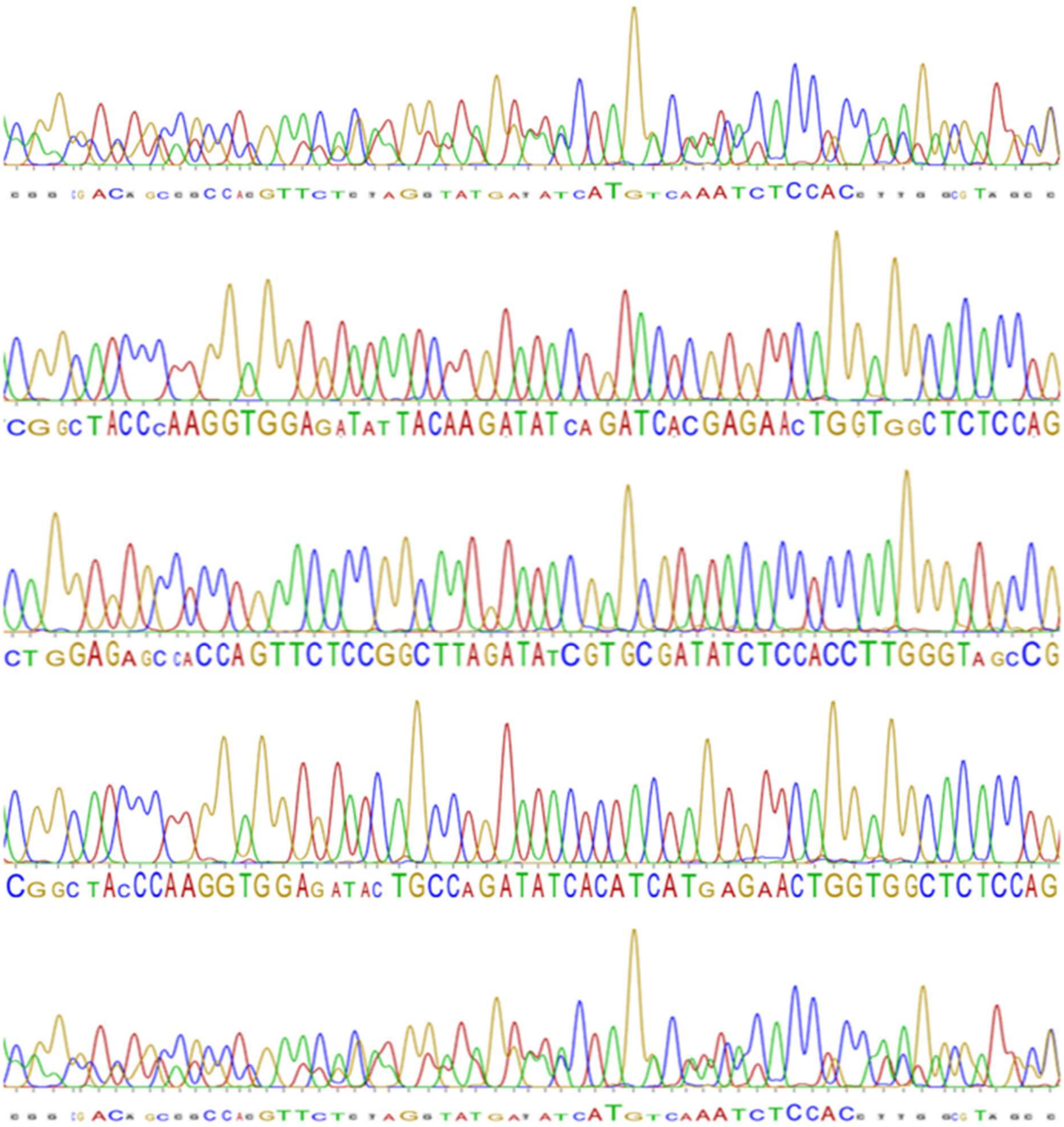

d-DNA 4-BamHI

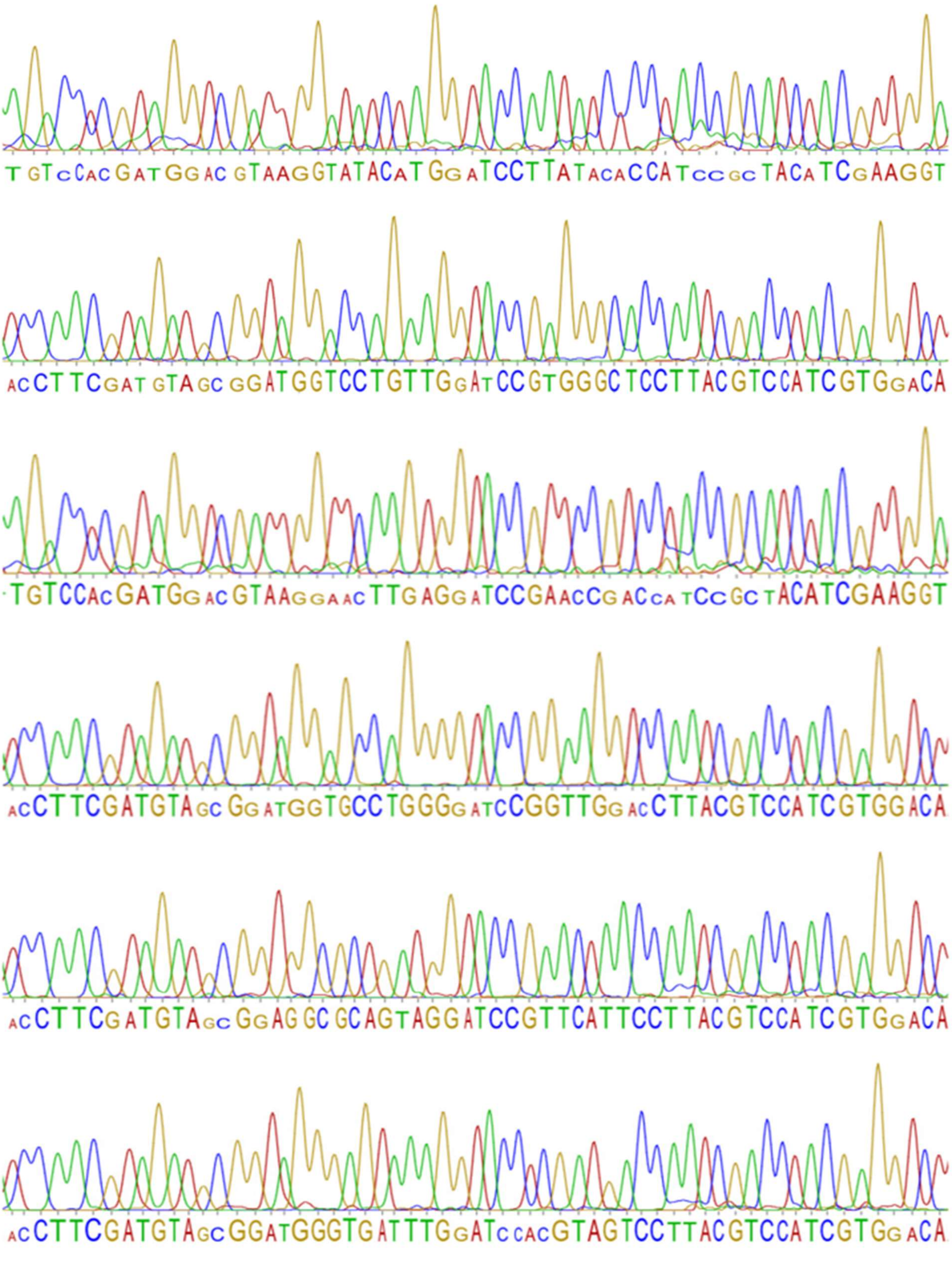
TGTCCACGATGGACGTAAGGTATACATGGATCCTTATACACCATCCGCTACATCGAAGGT
ACCTTCGATGTAGCGGATGGTCCTGTTGGATCCGTGGGCTCCTTACGTCCATCGTGGACA
TGTCCACGATGGACGTAAGGAACTTGAGGATCCGAACCGACCATCCGCTACATCGAAGGT
ACCTTCGATGTAGCGGATGGTGCCTGGGGATCCGGTTGGACCTTACGTCCATCGTGGACA
ACCTTCGATGTAGCGGAGGCGCAGTAGGATCCGTTCATTCCTTACGTCCATCGTGGACA
ACCTTCGATGTAGCGGATGGGTGATTTGGATCCACGTAGTCCTTACGTCCATCGTGGACA

d-DNA 4-BamHI

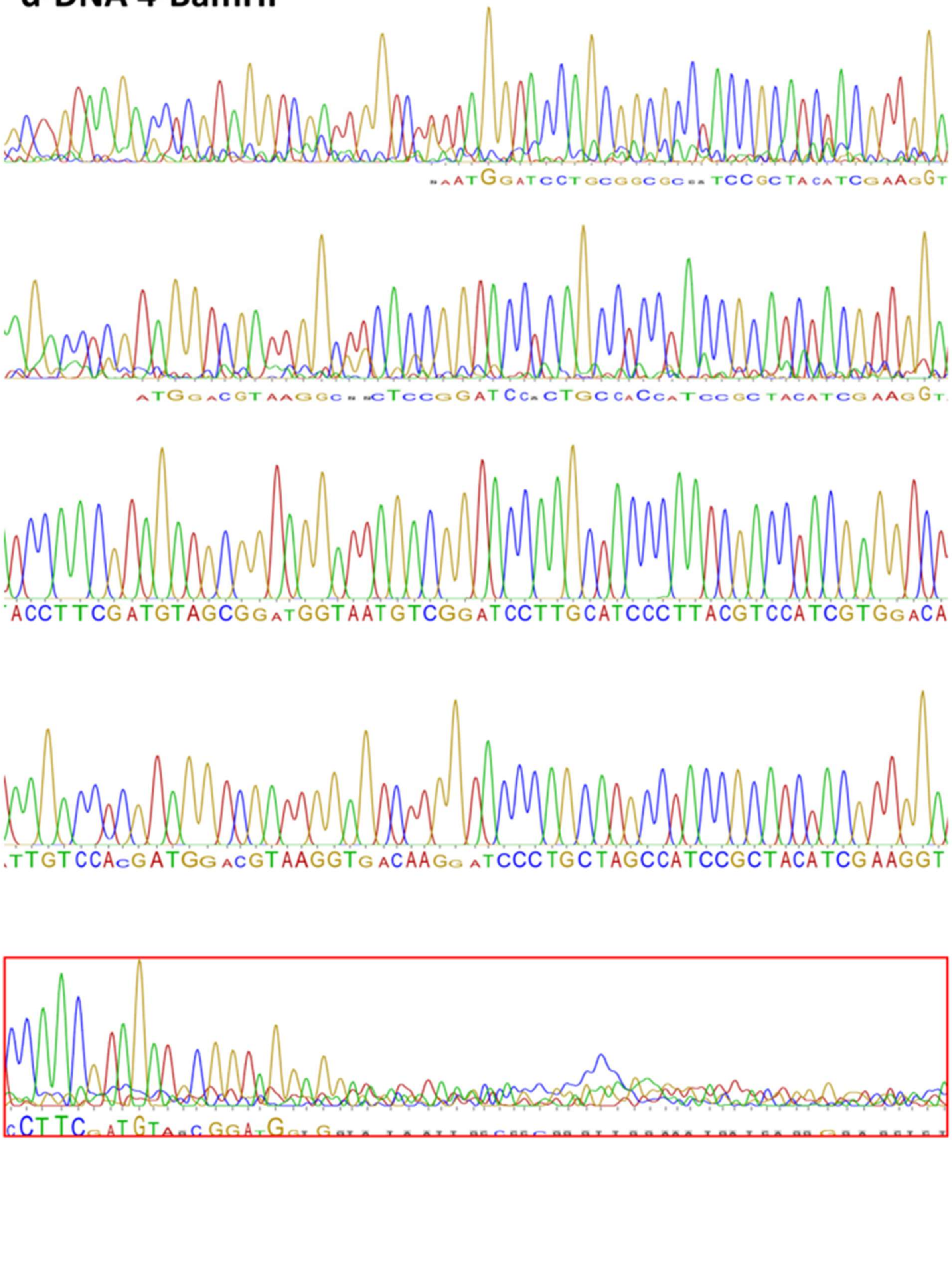

d-DNA 4-EcoRV

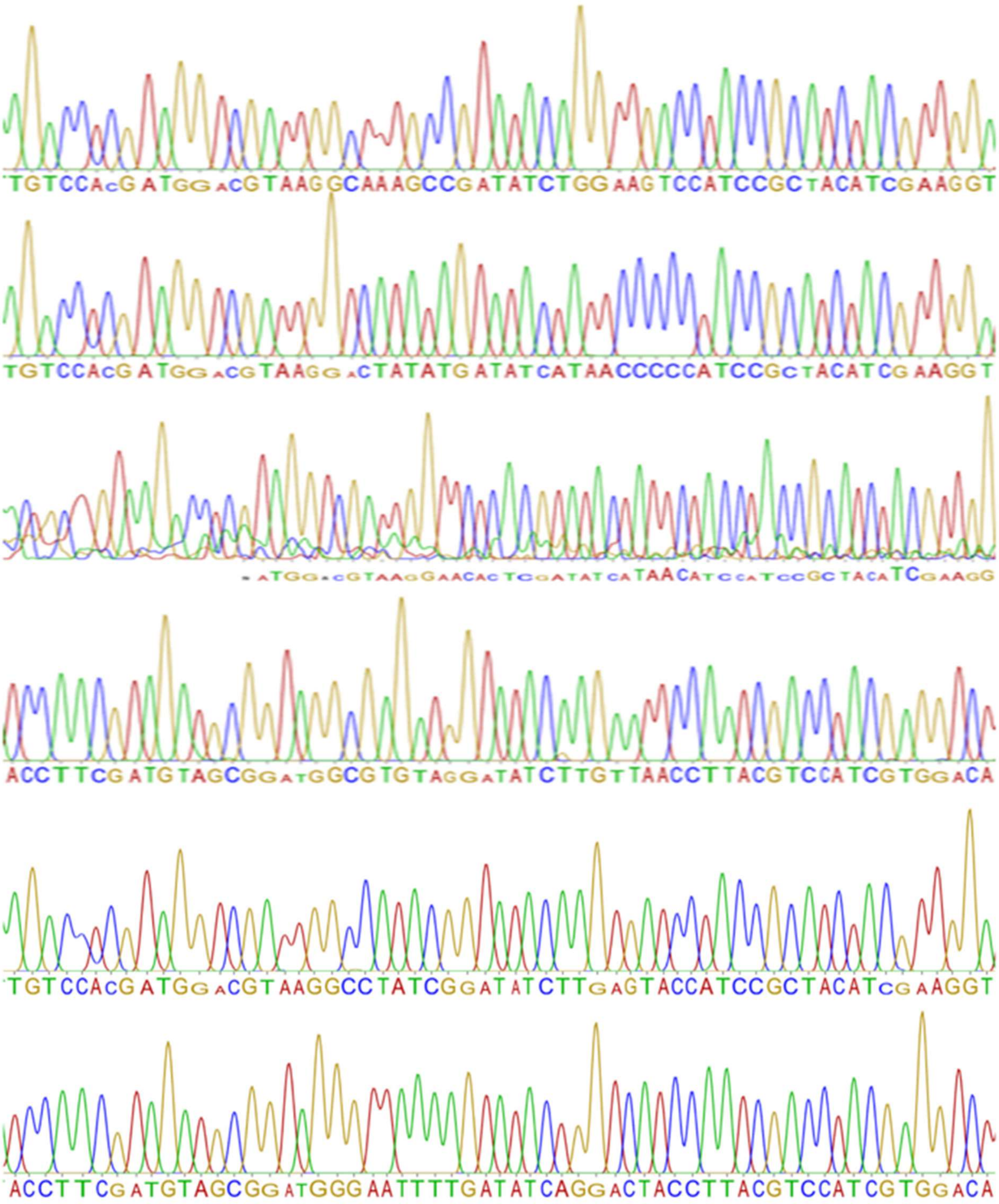

TGTCCACGATGGACGTAAGGCAAAGCCGATATCTGGAAGTCCATCCGCTACATCGAAGGT
TGTCCACGATGGACGTAAGGACTATATGATATCATAACCCCCATCCGCTACATCGAAGGT
-ATGG-CGTAAGGAACACTCGATATCATAACATCCATCCGCTACATCGAAGG
ACCTTCGATGTAGCGGATGGCGTGTAGGATATCTTGTTAACCTTACGTCCATCGTGGACA
TGTCCACGATGGACGTAAGGCCTATCGGATATCTTGAGTACCATCCGCTACATCGAAGGT
ACCTTCGATGTAGCGGATGGGAATTTTGATATCAGGACTACCTTACGTCCATCGTGGACA

d-DNA 4-EcoRV

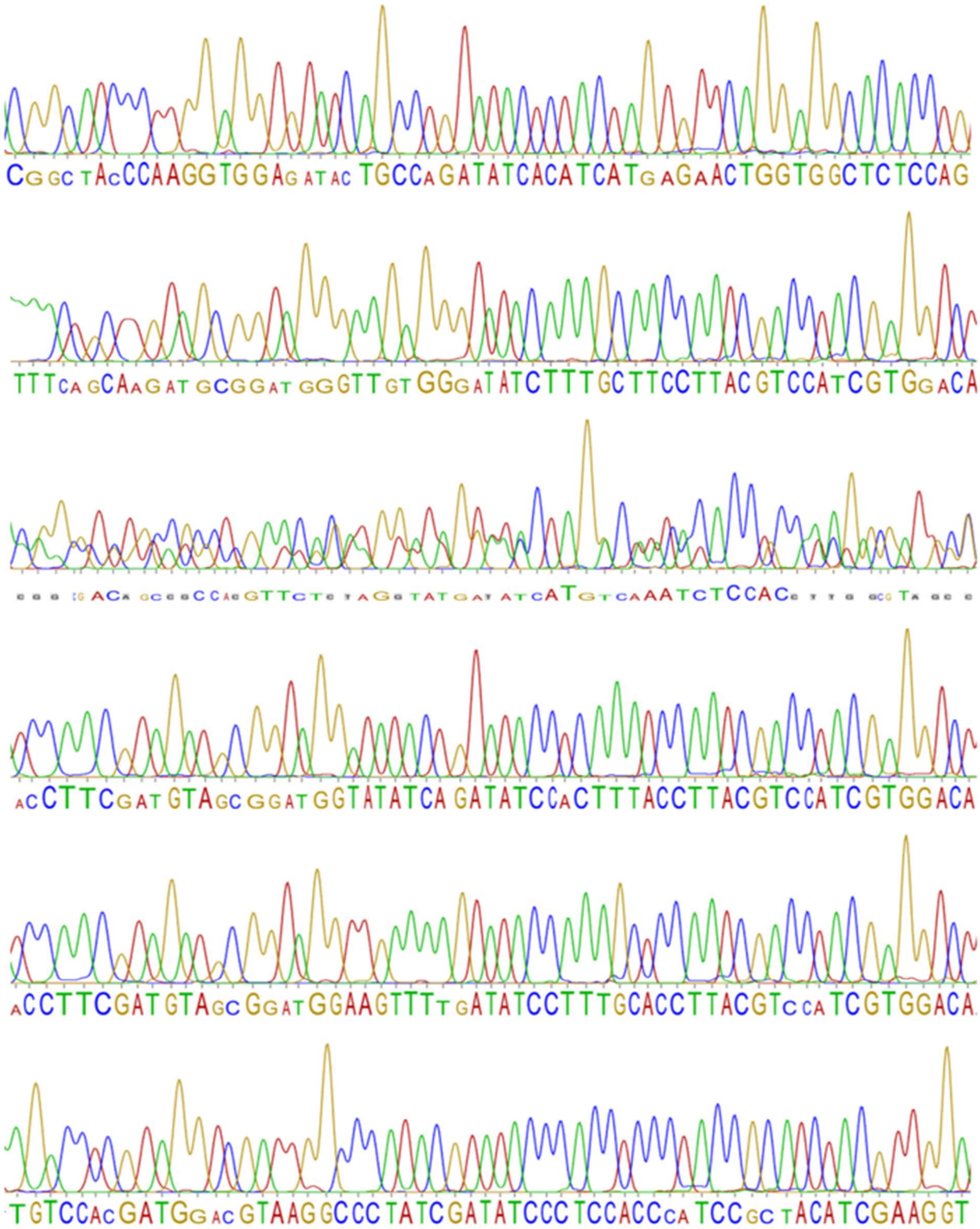
CGGCTACCCAAGGTGGAGATACTGCCAGATATCACATCATGAGAACTGGTGGCTCTCCAG
TTTCAGCAAGATGCGGATGGGTTGTGGGATATCTTTGCTTCCTTACGTCCATCGTGGACA
ACCTTCGATGTAGCGGATGGTATATCAGATATCCACTTTACCTTACGTCCATCGTGGACA
ACCTTCGATGTAGCGGATGGAAGTTTTGATATCCTTTGCACCTTACGTCCATCGTGGACA
TGTCCACGATGGACGTAAGGCCCTATCGATATCCCTCCACCCATCCGCTACATCGAAGGT

Figure S7. Sanger sequencing chromatogram of 50 plasmids isolated from 50 randomly picked colonies. Sequencing was performed using pJET1.2 forward sequencing primer. All chromatograms were sorted into each type of d-DNA (d-DNA 1 EcoRV, d-DNA 2 BamHI, d-DNA 2- EcoRV, d-DNA 3-BamHI, d-DNA 3-EcoRV, d-DNA 4- BamHI, and d-DNA 4-EcoRV).

2. Program of simulation for Figure 4f

```
#include <Carbon/Carbon.h>
#include <iomanip>
#include <ctime>
#include <iostream>
#include <fstream>
#include <sstream>
#include <math.h>
#include <cstdlib>
#include <ctime>
#include <string>
#include <stdio.h>
#include <stdlib.h>
using namespace std;


std::string rs;

int main()
{
    srand((unsigned)time(0));
    int i;
    int check;
    int n1, n2, n3;

    n2=1000;
    for (int n3 = 10000; n3 > 10; n3 = n3-200){
    n1=0;
    for (int r1 = 0; r1 < n2; r1++){
        check = 0;
        for (int r2 = 0; r2 < n3; r2++){
            i = (rand()%4)+1;
            if (i == 1) {rs += 'A';}
            if (i == 2) {rs += 'C';}
            if (i == 3) {rs += 'G';}
            if (i == 4) {rs += 'T';}

    }

    std::size_t found = rs.find("ACGTAT");
    std::size_t found2 = rs.find("TCAGTA");
    std::size_t found3 = rs.find("GTACGGTG");

    if
((found3!=std::string::npos)|(found2!=std::string::npos)|(found!=std::string:
:npos)){n1=n1+1;}
```

```
    rs.clear();
    }
    cout<<n1<< "\t" << n2 << "\t" <<n3<<"\n";
    }
    return 0;
}
```

3. Supplementary tables

Table S1 Sequence of oligonucleotides comprising DNA steganography

Oligonucleotides	Sequence (5' to 3')
i-DNA1	GACAATTCACACACGTCCGCAGTCTGACTGATCACTGGACATGAGATCGGAAGAGCGTCG
d-DNA1-SmaI	GACAATTCACACACGTCCGC NNNNNNN CCCGGG NNNNNNN ATGAGATCGGAAGAGCGTCG
d-DNA1- EcoRV	GACAATTCACACACGTCCGC NNNNNNN GATATC NNNNNNN ATGAGATCGGAAGAGCGTCG
i-DNA 2	CTCATCCGCTGTCTGTACCAATCTATGCCTGTATTAAAGCGACGACATCAACTCCAGGGT
d-DNA 2-EcoRV	CTCATCCGCTGTCTGTACCANNNNNNNGATATCNNNNNNNGACGACATCAACTCCAGGGT
d-DNA2-BamHI	CTCATCCGCTGTCTGTACCANNNNNNNGGATCCNNNNNNNGACGACATCAACTCCAGGGT
i-DNA 3	CGGCTACCCAAGGTGGAGATCGAACGAATCACTTCACTAGGAGAACTGGTGGCTCTCCAG
d-DNA 3-EcoRV	CGGCTACCCAAGGTGGAGATNNNNNNNGATATCNNNNNNNGAGAACTGGTGGCTCTCCAG
d-DNA 3-BamHI	CGGCTACCCAAGGTGGAGATNNNNNNNGGATCCNNNNNNNGAGAACTGGTGGCTCTCCAG
i-DNA 4	TGTCCACGATGGACGTAAGGCATCATGATCTCGCGTGTCTCCATCCGCTACATCGAA GGT
d-DNA 4-EcoRV	TGTCCACGATGGACGTAAGGNNNNNNNGATATCNNNNNNNCCATCCGCTAC ATCGAAGGT
d-DNA 4-BamHI	TGTCCACGATGGACGTAAGGNNNNNNNGGATCCNNNNNNNCCATCCGCTAC ATCGAAGGT

Table S2 Primer Sequence

Primers	Sequence (5' to 3')
key-1i-DNA-1-REV	CGACGCTCTTCCGATCTCAT
Key-1-i-DNA-FOR	GACAATTCACACACGTCCGC
Wrong key-1-R	GAGATCGGAAGAGCGTCG
Wrong key-1-F	TGGTCTCAGCCGCCCTAT
Key-1-i-DNA-2 REV	ACCCTGGAGTTGATGTCGTC
key-1-i-DNA-2 FOR	CTCATCCGCTGTCTGTACCA
Key-1-i-DNA-3 REV	CTGGAGAGCCACCAGTTCTC

key-1-i-DNA-3 FOR	CGGCTACCCAAGGTGGAGAT
Key-1-i-DNA-4 REV	ACCTTCGATGTAGCGGATGG
key-1-i-DNA-4 FOR	TGTCCACGATGGACGTAAGG
